# “DompeKeys”: a set of novel substructure-based descriptors for efficient chemical space mapping, development and structural interpretation of machine learning models, and indexing of large databases

**DOI:** 10.1186/s13321-024-00813-4

**Published:** 2024-02-23

**Authors:** Candida Manelfi, Valerio Tazzari, Filippo Lunghini, Carmen Cerchia, Anna Fava, Alessandro Pedretti, Pieter F. W. Stouten, Giulio Vistoli, Andrea Rosario Beccari

**Affiliations:** 1 EXSCALATE, Dompé Farmaceutici SpA, Via Tommaso de Amicis 95, 80123 Napoli, Italy; 2 Department of Pharmacy, University of Naples “Federico II”, Via D. Montesano 49, 80131 Napoli, Italy; 3 Dipartimento di Scienze Farmaceutiche, Università degli Studi di Milano, Via Mangiagalli, 25, 20133 Milano, Italy; 4 Stouten Pharma Consultancy BV, Kempenarestraat 47, 2860 Sint-Katelijne-Waver, Belgium

**Keywords:** Chemical space, SMARTS, Chemical pattern search, Scaffold analysis, Machine learning, Artificial intelligence, Drug design, Drug metabolism

## Abstract

The conversion of chemical structures into computer-readable descriptors, able to capture key structural aspects, is of pivotal importance in the field of cheminformatics and computer-aided drug design. Molecular fingerprints represent a widely employed class of descriptors; however, their generation process is time-consuming for large databases and is susceptible to bias. Therefore, descriptors able to accurately detect predefined structural fragments and devoid of lengthy generation procedures would be highly desirable. To meet additional needs, such descriptors should also be interpretable by medicinal chemists, and suitable for indexing databases with trillions of compounds. To this end, we developed—as integral part of EXSCALATE, Dompé’s end-to-end drug discovery platform—the DompeKeys (DK), a new substructure-based descriptor set, which encodes the chemical features that characterize compounds of pharmaceutical interest. DK represent an exhaustive collection of curated SMARTS strings, defining chemical features at different levels of complexity, from specific functional groups and structural patterns to simpler pharmacophoric points, corresponding to a network of hierarchically interconnected substructures. Because of their extended and hierarchical structure, DK can be used, with good performance, in different kinds of applications. In particular, we demonstrate how they are very well suited for effective mapping of chemical space, as well as substructure search and virtual screening. Notably, the incorporation of DK yields highly performing machine learning models for the prediction of both compounds’ activity and metabolic reaction occurrence. The protocol to generate the DK is freely available at https://dompekeys.exscalate.eu and is fully integrated with the Molecular Anatomy protocol for the generation and analysis of hierarchically interconnected molecular scaffolds and frameworks, thus providing a comprehensive and flexible tool for drug design applications.

**Supplementary Information:**

The online version contains supplementary material available at 10.1186/s13321-024-00813-4.

## Introduction

At the root of cheminformatics and computer-aided drug design, the capacity to encode molecular structures into a computer readable form represents a relevant need. According to their dimensionality, the molecular representations can be subdivided into: (i) one-dimensional (1D, e.g., alphanumeric strings), (ii) two-dimensional (2D, e.g., molecular graphs), and (iii) three-dimensional (3D, e.g., molecular coordinates). The SMILES (Simplified Molecular Input Line Entry System) notation is a very popular 1D representation, introduced in the 1980s [1], by which a molecule is represented as a simple sequence of characters with predefined atom ordering rules. Daylight uses an extension of SMILES called SMARTS to describe structure queries for searching chemical databases [2]. The IUPAC International Chemical Identifier (InChI) was introduced in 2000 and was designed as a strictly unique standard chemical identifier [3]. A compact hashed code was then derived from InChI (InChIKey).

Along with notations able to unambiguously describe entire molecular structures, there are many representations able to describe features and substructures included in a given molecule. Molecular fingerprints [4] are computationally efficient representations, in which structural features are encoded as bits in a bit string or counts in a count vector, thus capturing the main structural characteristics and chemical properties.

The MACCS keys encode the presence of predefined substructures into a vector of length 166 bits [5]. The extended connectivity fingerprints (ECFP) are not based on substructure dictionaries but perceive the presence of substructures around each atom in a molecule, using a hash function to store information for each atom’s neighborhood up to a predefined diameter [6].

Atom pair fingerprints encode molecular shape [7], and have been reported to be more suitable to represent large molecules, such as those exceeding the Lipinski limits [8].

In recent work, the atom-pair approach was combined with circular substructures to create a new descriptor, called MAP4 (MinHashed atom-pair fingerprint up to a diameter of four bonds), providing a unified description of molecules across different sizes and shapes [9].

In other work, neural network fingerprints were generated by training neural networks on target specific bioactivity datasets [10]. As initial case study, the generic features that are most common amongst kinase inhibitors (e.g., a hinge-binding motif), were considered. The best performing architecture was based on a Multilayer Perceptron (MLP) with the ECFPs as the input, trained for multitask classification (to predict the specific kinase target activity).

Very recently, functional-group-like structural fragments (FGSFs) were implemented as a set of predefined structural moieties commonly found in organic molecules, annotated with reactivity parameters, and successfully applied to toxicophore identification and machine learning applications [11].

Generally speaking, these representations are of pivotal importance for storing chemical structures and for utilizing chemical structural information in similarity/substructure searches, as well as for the construction of chemical space maps. The choice of the descriptor is critical to the success of a similarity search, because each descriptor focuses on different chemical properties. Also, the size of the bit space of the fingerprints was reported to have a significant effect on enrichments, that is, the ability to identify compounds with activity similar to a query molecule, with small bit spaces, such as 1024, resulting in collisions and in turn in a substantial reduction in enrichments compared to larger bit spaces [12]. Further benchmark studies on fingerprints performance reported that the differences in enrichment and the number of collisions observed in the earlier study [12] are likely due to the use of different hashing functions and different bit densities across the fingerprints used [13]. Consequently, descriptors devoid of possible biases caused by the fingerprint generation procedures are greatly needed.

Therefore, we set out to design a new set of descriptors. The goals of these descriptors are multifold:

1. They must adequately separate molecules in chemical space, which belong to different classes,
2. They must be suitable for the development of Machine Learning models and the interpretation of such models in terms that are meaningful to medicinal chemists,
3. They must provide key characteristics of individual molecules at a glance, and
4. Together with Dompé’s “Molecular Anatomy” [14], they must be able to efficiently index databases with more than 10s of trillions (10^13^) of chemical structures.

**Fig. 1:**
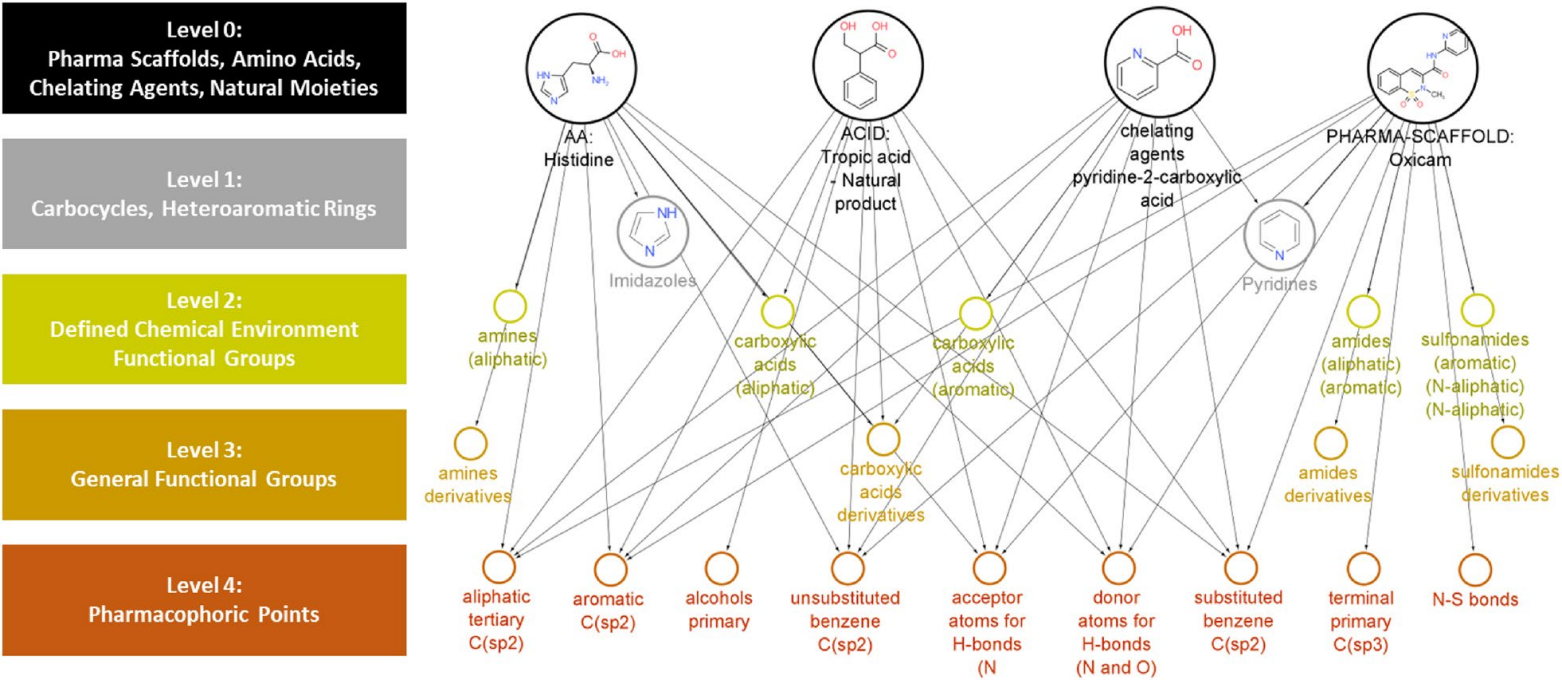
DompeKeys (DK): a new hierarchical substructure-based descriptor

In this work we describe the DompeKeys (DK), a new set of substructure-based fingerprint descriptors, which encode patterns of functional groups and chemical features contained in compounds of pharmaceutical interest, and we also report their performance in terms of the aforementioned goals (1) and (2). The DK system collects 1064 curated SMARTS strings, encoding chemical structures at different levels of complexity, from well-defined structural moieties, like amino acids or metal binders and tox alerts, to generic pharmacophoric features, like H-bond donor or acceptors. Each functional group is either encoded as is or includes additional information about its chemical environment, thus constituting a network of hierarchically interconnected substructures (Fig. 1). In addition, we developed a validation protocol to demonstrate the integrity and correctness of the DK formalism.

We demonstrate that DK are very well-suited to map the chemical space of databases of compounds that are different in terms of physicochemical properties. Moreover, we report the successful application of the DK for the prediction of compounds’ activities and metabolic reactions by machine learning (ML) models, showing that they quickly identify the key chemical moieties for biological activity. Additionally, we show how the DK can be extremely helpful in substructural search and pharmacophoric filtering, making simpler and faster to screen and prioritize compounds possessing functional groups relevant for a certain biological activity among millions of molecules.

The protocol to generate DK is freely available within the web interface https://dompekeys.exscalate.eu, where it is fully integrated with the Molecular Anatomy approach, an in-house developed method to analyze large datasets of molecules by organizing them into a multi-dimensional network of hierarchically interconnected molecular frameworks.

## Results and discussion

### Descriptor design

The DK, coded in the robust SMARTS language, are designed not only to describe simple functional groups but also to explore the chemical environment of each functional group, to search for fragments with specific reactivity or physicochemical properties or even structural toxicity alerts.

We collected a list of 1064 manually curated SMARTS strings, each one encoding chemical structures and functional groups defining different levels of complexity (from level 0 to 4, ranging from the highest to the lowest molecular complexity, respectively).

In detail, level 0 represents the highest level of molecular complexity, by including well-defined molecular structures, such as amino acids, natural products, drugs (Fig. 1). Level 1 features more specific representations than level 0, and contains mostly ring systems (such as pyridine, imidazole). Levels 2 and 3 describe the main functional groups, such as amines, amide groups, with the only difference that in level 2 we differentiate between the number and the nature of substituents, for instance if a given amine is primary, secondary or tertiary and also if the attached substituents are aliphatic or aromatic. As such, level 2 allows a more precise mapping of the chemical environment of a given functional group.

Finally, level 4 represents the simple atoms with specific properties, such as sp2 carbons or nitrogen atoms that can function as H-bond acceptor, from which we can derive simple pharmacophoric points. Taken together, the five levels make up a network of hierarchically interconnected substructures.

The DK were conceptualized following a knowledge-based approach: DK levels 4 through 2 were essentially hand-crafted, considering the most standard patterns and functional groups commonly found in organic drug-like molecules. Higher levels 1 and 0 were collected taking into account amino acids, structural fragments and scaffolds (e.g., heterocycles) derived from analysis of approved drugs (pharmascaffold), commercial and natural compounds libraries. In addition, patterns and annotations such as toxicophores or metal binders were also included, based on a combination of literature search [15, 16] and in-house expertise gained in the context of internal drug discovery projects. This hierarchical architecture makes DK able to capture key structural information in different types of applications, with the possibility to select even only subsets to be used on a case-by-case basis.

A practical example is shown in Table 1. The example compound tucidinostat, a potent and orally bioavailable Histone Deacetylase inhibitor, has been analyzed using the protocol to generate DK. The protocol allows any (medicinal and computational) chemist to easily and quickly gain insights about the molecular structure at different hierarchical levels, such as the main functional groups, whether it contains undesirable functionalities and even the presence of structural fragments annotated with a specific pharmacological activity. For instance, at level 0 a fragment essential for the chelation of metals the n-(2-aminophenyl)acetamide, is flagged.

**Table 1:** Key structural information for tucidinostat from DK mapping


DK-QueryMapped	Description	Hierarchy	Highlighted structure
[#6]-[CX3](-[N;H1]-[c]1:[c]:[c]:[c]:[c]:[c]1-[N;H2])=[OX1]	N-(2-aminophenyl)acetamide derivatives	Level 0 Chelating agent Complex fragment	
[nR1]1[cR1][cR1][cR1][cR1][cR1]1	Pyridines	Level 1 Carbocycles, heteroaromatic rings	
[C][CX3;!$(C(=O)[*][OH,SH,NH]);!$(C(=O)C(=O));!$(C(=O)(N)[O,N,S])](=[OX1])[NX3;H1;!$(N[*][OH,SH,NH]);!$(N[Cl,Br,I,F,N,S,O]);!$(N(C(=[O,S,N]))[CX3](=[N,S,O]))][C]	Secondary amides (aliphatic)/(n-aliphatic)/Defined chemical environment functional group	Level 2 Defined chemical environment functional group	
[c][CX3;!$(C(=O)[*][OH,SH,NH]);!$(C(=O)C(=O));!$(C(=O)(N)[O,N,S])](=[OX1])[NX3;H1;!$(N[*][OH,SH,NH]);!$(N[Cl,Br,I,F,N,S,O]);!$(N(C(=[O,S,N]))[CX3](=[N,S,O]))][c]	Secondary amides (aromatic)/(n-aromatic) Defined chemical environment functional group	Level 2 Defined chemical environment functional group	
[NX3;H2;!$(N-[N,O,S,Cl,Br,I,F]);!$(NC(=[N,O,S]))][c]	Primary amines (aromatic) Tox Alert	Level 2 Defined chemical environment functional group	
[Cl,Br,F,I][a;!r0]	X on aromatic ring	Level 2 Defined chemical environment functional group	
[CX3;!$(C(=O)[*][OH,SH,NH]);!$(C(=O)C(=O));!$(C(=O)(N)[O,N,S])](=[OX1])[NX3;!$(N[*][OH,SH,NH]);!$(N[Cl,Br,I,F,N,S,O]);!$(N(C(=[O,S,N]))[CX3](=[N,S,O]))]	Amides derivatives general functional group	Level 3 general functional group	
[NX3;H3,H2,H1,H0;!$(N-[N,O,S,Cl,Br,I,F]);!$(NC(=[N,O,S]))]	Amine derivatives	Level 3 general functional group	
[Cl,Br,F,I][[#6];!$(C(=O)]	X derivatives	Level 3 general functional group	
[CX3](=[A])(-[*!X1])-[*!X1]	Aliphatic tertiary C(sp2)	Level 4 Pharmacophoric points	
[cH0]	Substituted benzene C(sp2)	Level 4 Pharmacophoric points	
[c]	Aromatic C(sp2)	Level 4 Pharmacophoric points	
[!H0;#7,#8,#9]	Donor atoms for H-bonds (N and O)	Level 4 Pharmacophoric points	
[!$([#6,F,Cl,Br,I,o,s,nX3,#7v5,#15v5,#16v4,#16v6,* + 1,* + 2,* + 3])]	Acceptor atoms for H-bonds (N,O,F)	Level 4 Pharmacophoric points	

The same fragment is mapped also by level 3 as generic amine, and by level 2 as aromatic primary amine, also flagging a possible structural alert because of the presence of the aniline moiety. Finally, level 4 specifies the pharmacophoric points found, such as donor, acceptor, halogen, aromatic carbon. This analysis provides a comprehensive overview of the molecule’s chemical properties; the chemist is informed about the presence of possible toxicophores and functional groups annotated with a certain pharmacological activity, which helps the compound selection process.

To further verify the validity of the curated list of DKs, we developed a unit-testing protocol in Pipeline Pilot [17]. Specifically, we converted the DKs from level 0 and 1 to explicit connection tables in mol2 format and verified how the SMARTS string encoding each DK is able to exactly map the corresponding molecule, as well as fragments comprised into the molecule structure, without duplicating functional groups (Table 2). A second step of validation involved DKs corresponding to functional groups described by both a generic SMARTS string (level 3) and by more specific SMARTS accounting for different chemical environments. For each generic SMARTS from level 3, we considered all possible permutations, i.e., we substituted the free valence on the molecule with H, methyl and an aromatic ring; these latter were then converted in whole molecule and the substructure searches of both the generic (level 3) and the specific SMARTS (level 2) were performed to demonstrate that both queries are satisfied. As an example, Table 2 reports two molecules encoded by DK of level 0, namely the amino acid tryptophan and nicotinic acid, a natural product. However, they can also be mapped by DKs of level 1 encoding heteroaromatic rings. Therefore, query molecules can be retrieved at different search levels.

**Table 2:** Example molecules used for DK validation

Molecule	DK-Level0	DK-Level1	DK-Level2	DK-Level3
	AA: Tryptophan	Indoles	Carboxylic Acids aliphatic Primary Amine aliphatic	Carboxylic Acids derivatives Amine derivatives
	ACID: Nicotinic Acid—Natural product	Pyridines	Carboxylic Acids aromatic	Carboxylic Acids derivatives

Notably, DK include functional group descriptors at two different levels: in the first one, a given functional group is described by a generic SMARTS string encoding only its specific atoms and also excluding from its environment chemically invalid patterns; then, for the same functional group, more specific SMARTS strings are defined, considering the different classes of substituents (aromatic and/or aliphatic atoms). Table 3 reports an example of these SMARTS strings describing carbamate derivatives.

**Table 3:** Chemical environment of the functional groups (description levels 2 and 3)

Functional Group	DK
Carbamate derivatives	[OX2;!$(O[N,O,S,Cl,Br,I,F]);!$(O(C=[N,O,S])C=[N,O,S])][CX3](=[OX1])[NX3;!$(N[N,O,S,Cl,Br,I,F]);!$(N(C=[N,O,S])C=[N,O,S])]
Carbamates (o-aliphatic)	[CX3](=[OX1])([OX2;!$(O[N,O,S,Cl,Br,I,F]);!$(O(C=[N,O,S])C=[N,O,S])][C])[NX3;!$(N[N,O,S,Cl,Br,I,F]);!$(N(C=[N,O,S])C=[N,O,S])]
Carbamates (o-aliphatic)/(n-aliphatic)	[CX3](=[OX1])([OX2;!$(O[N,O,S,Cl,Br,I,F]);!$(O(C=[N,O,S])C=[N,O,S])][C])[NX3;!$(N[N,O,S,Cl,Br,I,F]);!$(N(C=[N,O,S])C=[N,O,S])][C]
Carbamates (o-aliphatic)/(n-aliphatic)/(n-aliphatic)	[CX3](=[OX1])([OX2;!$(O[N,O,S,Cl,Br,I,F]);!$(O(C=[N,O,S])C=[N,O,S])][C])[NX3;!$(N[N,O,S,Cl,Br,I,F]);!$(N(C=[N,O,S])C=[N,O,S])]([C])[C]
Carbamates (o-aliphatic)/(n-aromatic)	[CX3](=[OX1])([OX2;!$(O[N,O,S,Cl,Br,I,F]);!$(O(C=[N,O,S])C=[N,O,S])][C])[NX3;!$(N[N,O,S,Cl,Br,I,F]);!$(N(C=[N,O,S])C=[N,O,S])][c]
Carbamates (o-aliphatic)/(n-aromatic)/(n-aliphatic)	[CX3](=[OX1])([OX2;!$(O[N,O,S,Cl,Br,I,F]);!$(O(C=[N,O,S])C=[N,O,S])][C])[NX3;!$(N[N,O,S,Cl,Br,I,F]);!$(N(C=[N,O,S])C=[N,O,S])]([c])[C]
Carbamates (o-aromatic)	[CX3](=[OX1])([OX2;!$(O[N,O,S,Cl,Br,I,F]);!$(O(C=[N,O,S])C=[N,O,S])][c])[NX3;!$(N[N,O,S,Cl,Br,I,F]);!$(N(C=[N,O,S])C=[N,O,S])]
Carbamates (o-aromatic)/(n-aliphatic)	[CX3](=[OX1])([OX2;!$(O[N,O,S,Cl,Br,I,F]);!$(O(C=[N,O,S])C=[N,O,S])][c])[NX3;!$(N[N,O,S,Cl,Br,I,F]);!$(N(C=[N,O,S])C=[N,O,S])][C]
Carbamates (o-aromatic)/(n-aromatic)	[CX3](=[OX1])([OX2;!$(O[N,O,S,Cl,Br,I,F]);!$(O(C=[N,O,S])C=[N,O,S])][c])[NX3;!$(N[N,O,S,Cl,Br,I,F]);!$(N(C=[N,O,S])C=[N,O,S])][c]
Carbamates (o-aliphatic)/(n-aromatic)/(n-aromatic)	[CX3](=[OX1])([OX2;!$(O[N,O,S,Cl,Br,I,F]);!$(O(C=[N,O,S])C=[N,O,S])][C])[NX3;H0;!$(N[N,O,S,Cl,Br,I,F]);!$(N(C=[N,O,S])C=[N,O,S])]([c])[c]
Carbamates (o-aromatic)/(n-aliphatic)/(n-aromatic)	[CX3](=[OX1])([OX2;!$(O[N,O,S,Cl,Br,I,F]);!$(O(C=[N,O,S])C=[N,O,S])][c])[NX3;H0;!$(N[N,O,S,Cl,Br,I,F]);!$(N(C=[N,O,S])C=[N,O,S])]([C])[c]
Carbamates (o-aromatic)/(n-aromatic)/(n-aromatic)	[CX3](=[OX1])([OX2;!$(O[N,O,S,Cl,Br,I,F]);!$(O(C=[N,O,S])C=[N,O,S])][c])[NX3;!$(N[N,O,S,Cl,Br,I,F]);!$(N(C=[N,O,S])C=[N,O,S])]([c])[c]
Carbamates (o-aromatic)/(n-aliphatic)/(n-aliphatic)	[CX3](=[OX1])([OX2;!$(O[N,O,S,Cl,Br,I,F]);!$(O(C=[N,O,S])C=[N,O,S])][c])[NX3;H0;!$(N[N,O,S,Cl,Br,I,F]);!$(N(C=[N,O,S])C=[N,O,S])]([C])[C]
Primary amides (aliphatic)	[C][CX3;!$(C(=O)[*][OH,SH,NH]);!$(C(=O)C(=O));!$(C(=O)(N)[O,N,S])](=[OX1])[NX3;H2;!$(N[*][OH,SH,NH]);!$(N[Cl,Br,I,F,N,S,O]);!$(N(C(=[O,S,N]))[CX3](=[N,S,O]))]
Secondary amides (aliphatic)/(aliphatic)	[C][CX3;!$(C(=O)[*][OH,SH,NH]);!$(C(=O)C(=O));!$(C(=O)(N)[O,N,S])](=[OX1])[NX3;H1;!$(N[*][OH,SH,NH]);!$(N[Cl,Br,I,F,N,S,O]);!$(N(C(=[O,S,N]))[CX3](=[N,S,O]))][C]
Tertiary amides (aliphatic)/(aliphatic)/(aliphatic)	[C][CX3;!$(C(=O)[*][OH,SH,NH]);!$(C(=O)C(=O));!$(C(=O)(N)[O,N,S])](=[OX1])[NX3;H0;!$(N[*][OH,SH,NH]);!$(N[Cl,Br,I,F,N,S,O]);!$(N(C(=[O,S,N]))[CX3](=[N,S,O]))]([C])[C]
Tertiary amides (aromatic)/(aliphatic)/(aliphatic)	[c][CX3;!$(C(=O)[*][OH,SH,NH]);!$(C(=O)C(=O));!$(C(=O)(N)[O,N,S])](=[OX1])[NX3;H0;!$(N[*][OH,SH,NH]);!$(N[Cl,Br,I,F,N,S,O]);!$(N(C(=[O,S,N]))[CX3](=[N,S,O]))]([C])[C]
Tertiary amides (aliphatic)/(aliphatic)/(aromatic)	[C][CX3;!$(C(=O)[*][OH,SH,NH]);!$(C(=O)C(=O));!$(C(=O)(N)[O,N,S])](=[OX1])[NX3;H0;!$(N[*][OH,SH,NH]);!$(N[Cl,Br,I,F,N,S,O]);!$(N(C(=[O,S,N]))[CX3](=[N,S,O]))]([C])[c]
Secondary amides (aliphatic)/(aromatic)	[C][CX3;!$(C(=O)[*][OH,SH,NH]);!$(C(=O)C(=O));!$(C(=O)(N)[O,N,S])](=[OX1])[NX3;H1;!$(N[*][OH,SH,NH]);!$(N[Cl,Br,I,F,N,S,O]);!$(N(C(=[O,S,N]))[CX3](=[N,S,O]))][c]
Tertiary amides (aliphatic)/(aromatic)/(aromatic)	[C][CX3;!$(C(=O)[*][OH,SH,NH]);!$(C(=O)C(=O));!$(C(=O)(N)[O,N,S])](=[OX1])[NX3;H0;!$(N[*][OH,SH,NH]);!$(N[Cl,Br,I,F,N,S,O]);!$(N(C(=[O,S,N]))[CX3](=[N,S,O]))]([c])[c]
Primary amides (aromatic)	[c][CX3;!$(C(=O)[*][OH,SH,NH]);!$(C(=O)C(=O));!$(C(=O)(N)[O,N,S])](=[OX1])[NX3;H2;!$(N[*][OH,SH,NH]);!$(N[Cl,Br,I,F,N,S,O]);!$(N(C(=[O,S,N]))[CX3](=[N,S,O]))]
Secondary amides (aromatic)/(aliphatic)	[c][CX3;!$(C(=O)[*][OH,SH,NH]);!$(C(=O)C(=O));!$(C(=O)(N)[O,N,S])](=[OX1])[NX3;H1;!$(N[*][OH,SH,NH]);!$(N[Cl,Br,I,F,N,S,O]);!$(N(C(=[O,S,N]))[CX3](=[N,S,O]))][C]
Tertiary amides (aromatic)/(aliphatic)/(aromatic)	[c][CX3;!$(C(=O)[*][OH,SH,NH]);!$(C(=O)C(=O));!$(C(=O)(N)[O,N,S])](=[OX1])[NX3;H0;!$(N[*][OH,SH,NH]);!$(N[Cl,Br,I,F,N,S,O]);!$(N(C(=[O,S,N]))[CX3](=[N,S,O]))]([C])[c]
Secondary amides (aromatic)/(aromatic)	[c][CX3;!$(C(=O)[*][OH,SH,NH]);!$(C(=O)C(=O));!$(C(=O)(N)[O,N,S])](=[OX1])[NX3;H1;!$(N[*][OH,SH,NH]);!$(N[Cl,Br,I,F,N,S,O]);!$(N(C(=[O,S,N]))[CX3](=[N,S,O]))][c]
Tertiary amides (aromatic)/(aromatic)/(aromatic)	[c][CX3;!$(C(=O)[*][OH,SH,NH]);!$(C(=O)C(=O));!$(C(=O)(N)[O,N,S])](=[OX1])[NX3;H0;!$(N[*][OH,SH,NH]);!$(N[Cl,Br,I,F,N,S,O]);!$(N(C(=[O,S,N]))[CX3](=[N,S,O]))]([c])[c]

This design feature of the DK allows mapping on each compound, both the presence of a generic functional group and its specific environment, to better evaluate the similarity between molecules. In contrast, the similarity of molecules containing the same functional group, but different substituents would be either overestimated by using only the descriptors of functional groups or underestimated by considering only descriptors focused on the surrounding atoms. Moreover, different description levels of DK could be useful when a given functional group or the specific fragment, of which it is part, should be recognized.

### Chemical space analysis

**Fig. 2:**
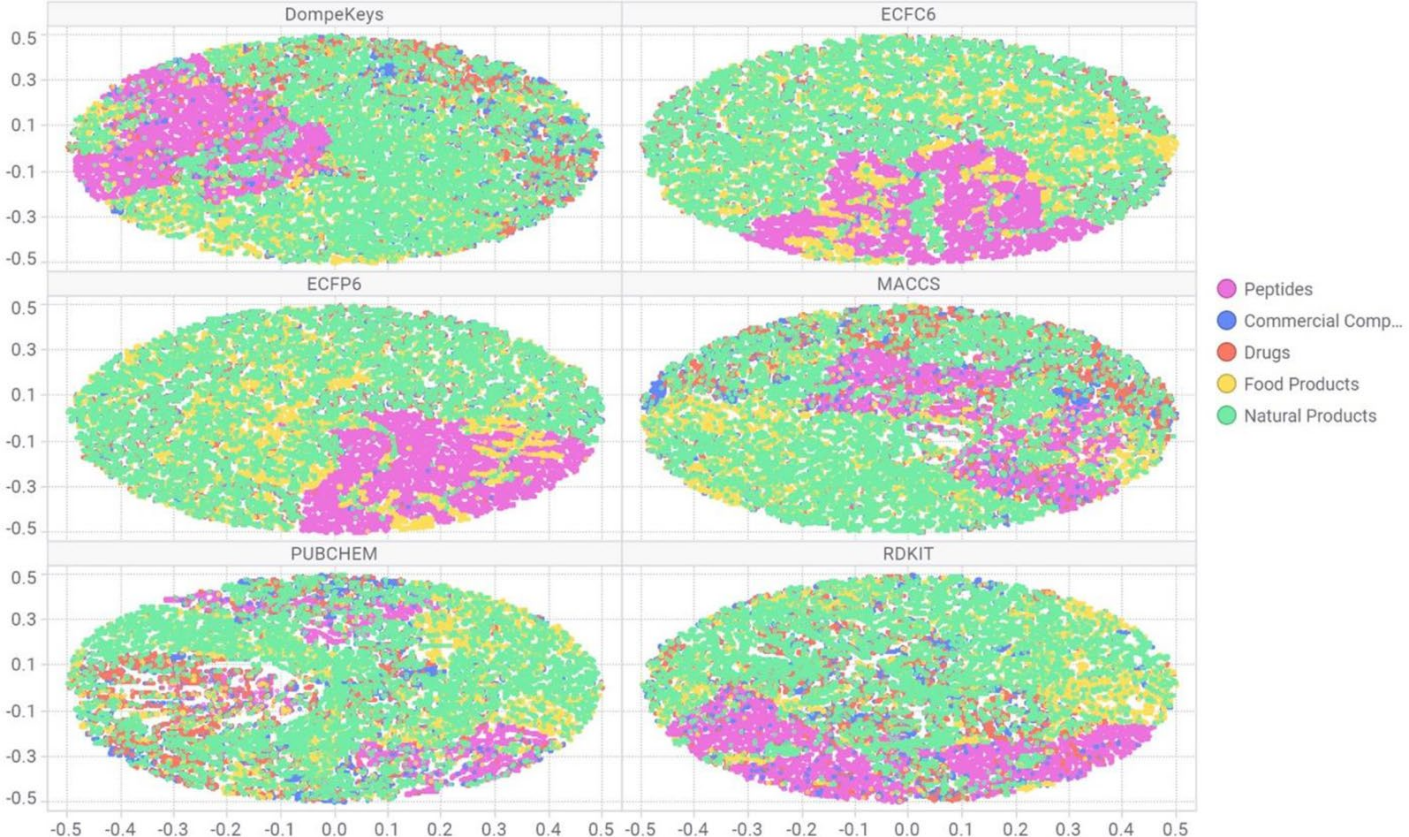
Chemical space analysis of structurally diverse libraries (drugs, peptides, natural products, food products, commercial compounds) by means of TMAP using DK in comparison with other descriptors

A detailed comparison of the chemical space covered by structurally diverse libraries of compounds (as described in the Materials and Methods section) was performed by means of Tree-MAP algorithm (TMAP) [18], which recently proved to have superior interpretability and discriminative power compared to other well-known methods such as t-SNE and SOM. Figure 2 reports the TMAP plots to compare the capability of the DK descriptor in classifying libraries with specific structural characteristics, with respect to other structural fingerprints, such as MACCS, ECFC6, ECFP6, PubChem and RDKit. In particular, the DK descriptors are able to better cluster the peptide chemical space, similarly to the ECFP6 and ECFC6. Moreover, DK are also able to better group and highlight the drugs class, which almost disappears or forms a very widely spread cluster in the maps based on other descriptors. Also, there is a certain overlap between the commercial compounds, the drugs and the natural products in the DK TMAP, which is to be expected because of the intrinsic similarities between such collections. In fact, several drugs and natural compounds are also commercially available, and several food products can be classified also as natural compounds.

These results can be explained by the presence of several SMARTS that encode common functional groups, which occur within these classes of compounds.

**Fig. 3:**
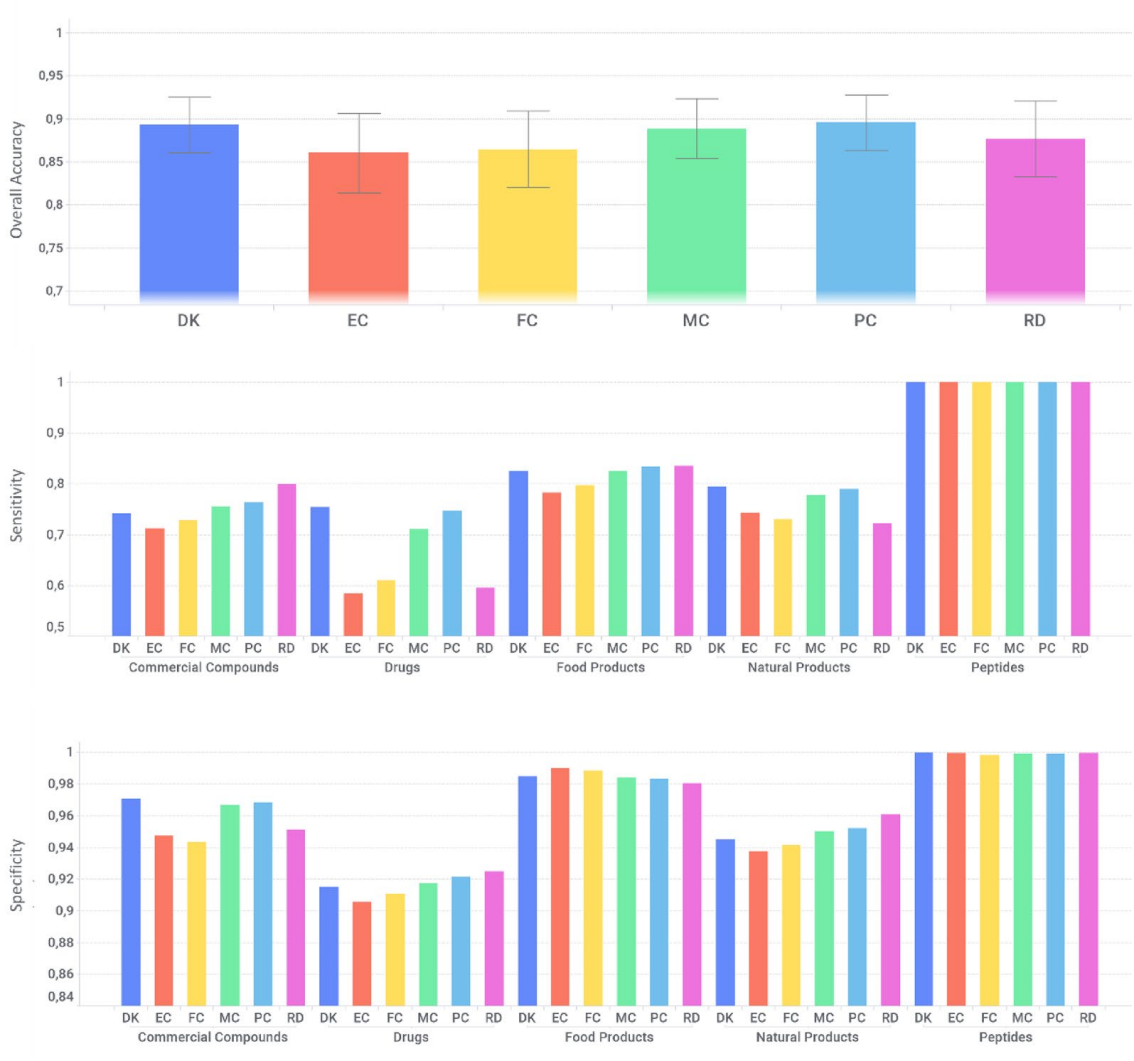
Bar chart representation for overall accuracy, sensitivity and specificity for the considered descriptors over the five chemical library classes. Performances are computed in external validation. Where: *DK* DompeKeys, *EC* extended connectivity, *FC* extended connectivity feature invariant, *MC* MACCS keys, *RD* RDKit, *PC* PubChem 881

The TMAP analysis essentially involves qualitative, visual inspection. To quantify the ability of DK to correctly classify the chemical collections, a multi-class classification model was developed. (see Additional file 1: Table S2 and Fig. 3). Overall, all descriptor spaces showed good discriminative power, especially for the peptides (SE = 0.99–1.0) and food products (SE = 0.89–0.90). This is expected as such chemical classes have some distinctive chemotypes which are effectively encoded by employed descriptors. A drop in performance can be seen when considering the class of the drugs, with sensitivity ranging from 0.58 (ECFP) to 0.75 (DK) and rather low precision values (around 0.1). This is due to the fact that commercial and drug compounds are often misclassified, as there is a strong overlap of chemotypes between these two chemical classes.

DK showed a good performance in discriminating chemical classes (overall accuracy = 0.89), on par with other popular descriptor spaces (PubChem, MACCS, RDKit, ECFP6 and ECFC6 scored 0.89, 0.88, 0.87, 0.86 and 0.85, respectively), which supports their utility for ligand-based virtual screening purposes.

Strikingly, the DK and PubChem 881 showed better sensitivity in classifying the drugs class in comparison with the other descriptors (Fig. 3). This finding suggests that such descriptors, based on pre-defined fragments, are able to perceive the most important aspects of compounds’ structures that have a crucial role for classification and retrieval. In the case of DK, whose fragments have been defined a priori with a high degree of coverage of functional groups and heterocycles present in drugs (i.e., pharmascaffold), they could form a “more compact” descriptor space, because the fragments are being precisely represented and might have an advantage over the descriptors generated automatically from the dataset, which might loss some chemical information.

### Ligand-based classification models

**Fig. 4:**
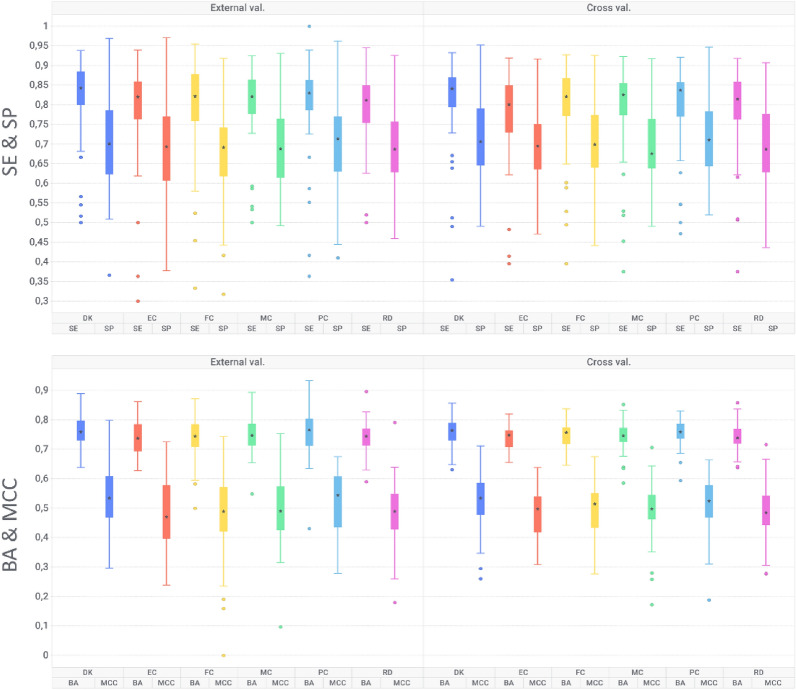
Box plot representation for balanced accuracy (BA), Matthews’s correlation coefficient (MCC), sensitivity (SE) and specificity (SP) evaluated in external and internal validation for the considered fingerprint types over the 46 modelled datasets. Where: *DK* DompeKeys, *EC* extended connectivity, *FC* extended connectivity feature invariant, *MC* MACCS keys, *RD* RDKit, *PC* PubChem 881

In addition to chemical space mapping, DK are also intended for the development of machine learning models to predict the inhibitory activity against biological targets. For this purpose, we constructed a curated dataset from ChEMBL, comprising compounds with inhibitory activity against 46 targets that are relevant for toxicity profiling (see Materials and Methods section and Additional file 3 for more details). Figure 4 depicts the model performance averaged over the 46 modelled datasets. Overall, all employed descriptors showed comparable performances, with an average BA of 0.74 (SD = 0.01), MCC of 0.48 (SD = 0.02), SE of 0.78 (SD = 0.01) and SP = 0.69 (SD = 0.01). DK exhibited performances at least as good as all other descriptor spaces, which underlines their power in encoding key molecular features related to biological activity. Moreover, DK scored the best performance in terms of MCC in 18 out of 46 datasets, followed by 14, 7, 7, 5 for ECFC6, ECFP6, RDKIT, PubChem and MACCS. In terms of SE and SP there are some differences for some specific datasets (see Additional file 1: Table S3). For instance, for the target EDNRA (Endothelin-1 receptor), DK, together with MACCS and ECFC6, scored the highest sensitivity at the expense of a much lower specificity (SE = 0.84, SP = 0.50) compared to the other descriptors (SE = 0.67–0.78, SP = 0.4–0.67).

When the learning task is related to chemical structures, a single molecular descriptor rarely produces the best performance in all case studies, as each descriptor space encodes for its own specific chemical moieties. A possible strategy to overcome individual-descriptor limitations is to construct an ensemble of multiple models trained on different descriptor spaces [19].

We also investigated the influence of the different levels of DK on the model’s discriminative power by rebuilding the ligand-based models using only specific DK levels. The worst performance (MCC = 0.40) is associated to level 4 DK (Additional file 1: Table S4) and significantly improves (*p* < 0.05) with the inclusion of higher levels DK (levels 0, 1 and 2). The highest performance is obtained by including all DK levels (MCC = 0.54) which supports the descriptor’s levels complementarity and synergies.

Hereafter, we calculated the frequency distribution of DK among the same dataset of ChEMBL compounds and reported the results in Additional file 1: Figure S2. As expected, the most frequently occurring DK are those belonging to level 4, i.e. the simple functional groups such as aromatic carbons, rings count, H-bond acceptors and H-bond donors. Moreover, there is a notable hotspot of amines, ethers and halogen-containing compounds, which are important reactive groups for drug-like molecules synthesis. Lastly, the most common heterocycles found were: piperidines, imidazoles, indoles and pyridines. Less represented DK (with a frequency value less than 5000) were grouped and shown in a single column labeled as “other” (Additional file 1: Figure S2).

In the search of structural features determining accurate predictions within the 46 modelled datasets, we extracted one of the protein targets for which the classification model performed particularly well, namely hERG (human ether-à-go-go-related gene, Uniprot ID: Q12809). The DK and PubChem are the top performing descriptors at modelling this drug-target interaction witch MCC of 0.51 and 0.52, respectively. The improvement with other descriptor types is notable (EC, FC, MC and RD MCC values are < 0.43), mainly due to misclassification of true negatives.

**Fig. 5:**
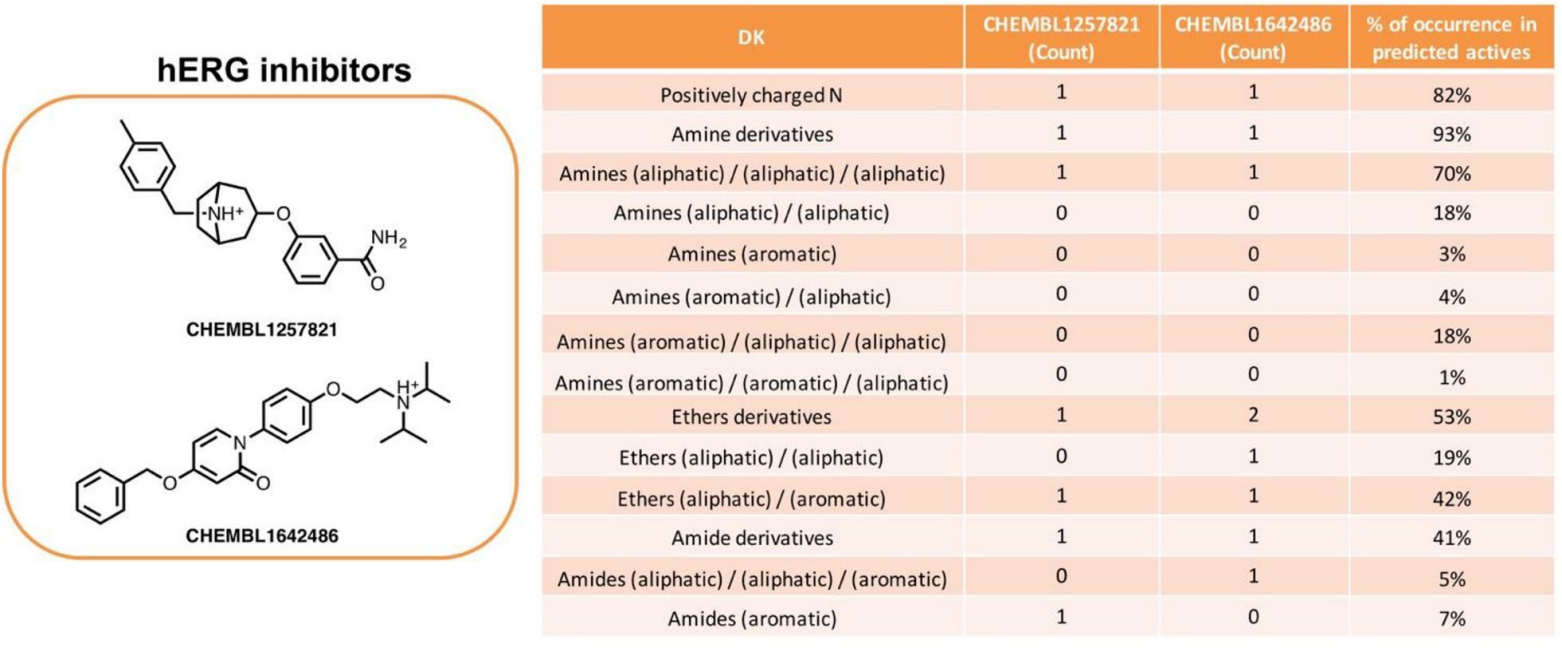
Mechanistic interpretation of most represented DK in hERG inhibitors, mapped on two example ligands

Subsequently, we pooled the most representative DK (i.e., the DK most frequently occurring in predicted actives as well as in correctly predicted actives), which were then mapped on two example ligands (Fig. 5). The full list of DK and their frequencies among hERG inhibitors is provided in Additional file 6. Besides the DK encoding very general substructures, such as aromatic rings or carbon chains, and thus occurring multiple times within a given active ligand, we were able to quickly identify specific functional groups having a key role on ligands’ activity against a given target.

With regard to hERG, we could identify some “privileged” DK such as amine derivatives (93%) and also ethers (53%) and amides (41%). Interestingly, a high percentage of the predicted actives feature a positively charged nitrogen (82%). This is consistent with the aliphatic tertiary amines being the most represented group within the active ligands (70%): in fact, it is known that this class of amines are protonated at physiological pH. In contrast, amine derivatives with aromatic substituents are less represented. Another interesting feature is the class of aliphatic/aromatic ethers, as hERG ligands are also characterized by bulky and aromatic scaffolds. It is worth noting that such functional groups can occur within a molecule structure multiple times; for instance, compound CHEMBL1642486 features two substituted ether groups: one “aliphatic/aliphatic” and one “aliphatic/aromatic”, with the percentages shown referred to the count of each functional group within the active ligands. Thus, the count of DK of level 2 (specific classes of ether derivatives) should not be summed up and compared to the count of DK level 3 (general ether derivatives).

The identified features are consistent with a common “hERG pharmacophore models” reported in literature, involving a basic moiety, playing an important role for the binding to hERG channel, and aromatic rings able to form π-stacking or hydrophobic interactions within the hERG channel cavity [20]. Hence, the hierarchically interconnected levels of DK allow quick perception of structural moieties that possess key roles in a ligand’s activity and can also be helpful in model interpretation. To further support our findings, we built a decision tree using DK of levels 2 and 3 as descriptors to analyze the dataset of hERG inhibitors. Notably, when employing the more general descriptor, namely DK level 3, defining, for example, amides, amines and ethers derivates, the model correctly classified only 31% of true active compounds. In contrast, when including a more precise amine representation (defining the substitution levels and the nature of substituents), encoded by DK level 2, the percentage of true positives greatly increased to 74%, suggesting that more “fine-graded” DK descriptors are truly able to capture meaningful structure–activity relationships. A graphical representation of extracted rules is depicted in Additional file 1: Figure S1.

Taken together, our results suggest that ML models for activity profiling based on DK showed performances as good as the models based on other popular 2D molecular descriptors; however, DK provide a more immediate overview of the peculiar structural features, allowing to quickly derive meaningful structure–activity relationships for the analyzed datasets.

### Drug design applications

Molecular similarity and pharmacophore modeling are frequently used approaches in the ligand-based drug design process. By using the molecular fingerprints of known ligands, databases can be screened to find similar molecules. Common structural features of ligands can be found using pharmacophore modeling, which can then be used to virtual screen for molecules with these features. The DK were designed to recognize not only simple functional groups but also fragments that are essential for a molecule’s activity against a specific target. They can therefore be useful in substructure searches, but they can also act as a pharmacophoric filter. Databases and libraries of trillions of compounds can be quickly queried to select compounds for acquisition and testing.

Moreover, mapping the chemical neighbor of a functional group, DK are also able to predict its reactivity. In particular DK representing pharmacophoric points, can be considered as atom typing descriptors and then used in predictive models of metabolic reaction occurrence, representing the simplest way to represent knowledge-based metabolic rules.

### Case Study 1: identification of HDAC7 inhibitors

In order to demonstrate the ability of DK to describe, in great detail, functional groups and chemical moieties, in particular identifying those responsible for a specific biological activity, we present, as case study, a screening campaign aimed at the identification of HDAC7 inhibitors, comparing the results, in terms of success rate, between a random library of 26,092 commercial compounds and an its targeted subset based on DK substructure selection. Histone deacetylases (HDACs) are key regulators of gene expression in cells and have been investigated as important therapeutic targets for cancer and other diseases [21]. Different subtypes of HDACs appear to play various roles in the cells and are associated with specific diseases. Therefore, substantial effort has been made to develop subtype selective HDAC inhibitors. The random library of 26,092 compounds was assembled with the aim to repurpose existing commercially available compounds as HDAC inhibitors. Out of the 26,092 compounds screened in HDAC7 enzymatic assay, 201 turned out to be active with a percent inhibition greater than 33%, corresponding to a success rate of 0.77%. The compounds were stratified in different activity classes according to their percent inhibition of HDAC7 activity obtained at 10 μM inhibitor concentration (Additional file 1: Table S5).

By applying a knowledge-based approach, codifying the known information related to the zinc binder functional group characteristic of HDAC inhibitors through DK, we could more easily prioritize compounds from the random library, thus increasing the success rate.

For this purpose, we prepared a list of 40 DK (with some examples reported in Table 4) exhaustively identifying all possible known metal binders of metalloproteases. Then, we used this list of 40 SMARTS strings, encoding the metal binder fragments, as a substructure filter against all the HDAC inhibitors retrieved from the Clarivate’s Cortellis database (800 molecules).

**Table 4:** Example HDAC inhibitors and their metal binder groups mapped by DK

HDAC inhibitors	DK mapped	Chemical name
	[#6]-[CX3](-[N;H1]-[c]1:[c]:[c]:[c]:[c]:[c]1-[N;H2])=[OX1]	n-(2-aminophenyl)acetamide
	[CX4][CX3](=[OX1])[NX3][OH]	Hydroxylamide aliphatic
	[S,O]=[#6]1-,:[#6]([OH])=,:[#6]-,:[#8]-,:[#6]=,:[#6]-,:1 c1ccc(c(c1)[OH])[OH] [OX2;H1;!$(OC(=[N,O,S]));!$(O[N,O,S,Cl,Br,F,I,P,B])]	3-hydroxy-4H-pyran-4-one benzene-1,2-diol hydroxil groups

We highlighted the DK that have been identified in the dataset compounds (Table 5) and used them for filtering the random library of 26,092 compounds. The recognized structures are not only chemical functional groups but also fragments (consisting of several connected atomic groups), able to bind metals (2-hydroxybenzoic acid, benzene-1,2-diol etc.).

**Table 5:** HDAC inhibitors DK used for the substructure filter

Chemical name	DK
n-(2-aminophenyl)acetamide	[#6]-[CX3](-[N;H1]-[c]1:[c]:[c]:[c]:[c]:[c]1-[N;H2])=[OX1]
Aryl sulphonamide	c1ccc(cc1)[Sv6X4](=[OX1])(=[OX1])[NX3H2,NX3H1]
Hydroxylamide aliphatic	[CX4][CX3](=[OX1])[NX3][OH]
3-hydroxy-4H-pyran-4-one	[S,O]=[#6]1-,:[#6]([OH])=,:[#6]-,:[#8]-,:[#6]=,:[#6]-,:1
Benzene-1,2-diol	c1ccc(c(c1)[OH])[OH]
Hydroxil group	[OX2;H1;!$(OC(=[N,O,S]));!$(O[N,O,S,Cl,Br,F,I,P,B])]
Quinolin-8-ol	c1ccc([OH,SH])c2c1cccn2
Hydroxylamide aromatic	[c][CX3](=[OX1])[NX3][OH]
2-methoxyphenol	c1ccc(c(c1)[OH,SH])[OX2][CX4]
2-hydroxybenzoic acid	[OH]c1ccccc1[CX3](=[OX1])[OH,NH2]
Pentane-2,4-dione	C[CX3](=[OX1])[CX4][CX3](=[OX1])C

The random library was further reduced to 2176 chemical entities, 54 of which turned out to be true actives, Table 6 reports some examples. The hit rate thus increased from 0.77 to 2.5%.

**Table 6:** Examples of molecules retrieved in the random library

Active molecules from random library	DK mapped	Chemical name
	[CX4][CX3](=[OX1])[NX3][OH]	Hydroxylamide aliphatic
	c1ccc(c(c1)[OH])[OH]	Benzene-1,2-diol

In addition, we performed an unbiased similarity search using the binary version of DK as fingerprint and comparing the results with ECFP6 and MACCS. Using the DK entire set of descriptors without prior knowledge, the success rate stands at 1.78%, 1.95% for MACCS and 1.66% for ECFP6. The combination of multiple descriptors led to a worsening of the result, considerably expanding the range of false positives. These findings further confirm the versatility of DK.

Thus, DK were able to exhaustively describe the chemical space of metal-binding fragments and to recognize, in the targeted library, the required moieties for the HDAC7 inhibition, also through an unbiased similarity search approach. This approach could be useful for further steps, such as focusing on selecting analogs with similar structural features.

### Case Study 2: Prediction of drug metabolism and toxicity

Previous studies have demonstrated that atom typing can be successfully utilized to predict the metabolic reactions a given substrate can undergo as well as the atom(s) undergoing the predicted reactions. The success of atom typing comes as no surprise, when considering that several predictive methods were based on a set of knowledge-based metabolic rules and atom typing can be seen as the simplest way to translate these rules in computationally tractable descriptors.

To evaluate their performance, the DK were used to predict the occurrence of three conjugation reactions, which play a key role in determining the drug toxicity by reducing the formation of reactive electrophilic metabolites (namely the conjugations with glucuronic acid, the sulfate anion and glutathione). Moreover, they were also utilized to predict the mutagenicity which is often also caused by the formation of reactive species. In detail and for each reaction, the analysis was based on a dataset with equal numbers of known substrates and non-substrates. The datasets were generated based on the MetaQSAR database [22] focusing on first-generation metabolic reactions and considering the molecules in their ionized state. Our study entailed a comparison of results obtained with DK and Kier-Hall E-state atom types, respectively. Conceivably, better results might be obtained by considering additional atom types and/or fingerprints. However, such an extended comparative analysis goes beyond the scope of this study and the comparison here was focused on the Kier-Hall E-state atom types, since they had proven to be satisfactory in published predictive models that were based on the same MetaQSAR datasets [23, 24].

Table 7 shows the performances of the classification models as obtained by Random Forest (RF) algorithm based on the two sets of descriptors for the three considered conjugations. The obtained performances underline the greater ability of the DK in encoding the substructures involved in the considered metabolic reactions. Conceivably, the enhancement is rather limited for the cases in which the KH atom types provided remarkable results (as seen in sulfonation). Gratifyingly, the enhancement increases in the cases in which KH atom types afforded poorer results suggesting that DK are particularly effective in the most challenging conditions.

**Table 7:** Performances of the classification models obtained by Random Forest (RF) algorithm based on the two sets of descriptors for the three considered conjugations

Reaction	Features	Recall (BA)	MCC	Specificity (SP)
Sulfonation	DK	0.89	0.79	0.90
	KH	0.87	0.75	0.89
Glucuronidation	DK	0.76	0.52	0.75
	KH	0.72	0.44	0.73
Reaction with glutathione	DK	0.81	0.62	0.78
	KH	0.78	0.56	0.75
Mutagenicity	DK	0.84	0.67	0.85
	KH	0.82	0.64	0.82

To better appreciate the DK enhanced ability to capture the reactive moieties, feature importance analysis was performed. As expected, sulfonation and glucuronidation share the most important features which correspond to aromatic and aliphatic hydroxyl groups as well as to aromatic rings. Nevertheless, the two types of hydroxyl groups play different roles in the two biotransformations since aromatic hydroxyl functions play a more relevant role in sulfonation, while aromatic and aliphatic hydroxyl groups show comparable relevance for glucuronidation. This difference is reflected in the other selected descriptors since DK encoding for rings, heterocycles and, in particular, N-containing heterocycles are included only in the model to predict sulfonation. In contrast, methyl groups, carboxylic acids, aliphatic chains play a favorable role in determining glucuronidation.

Conceivably, the reaction with glutathione depends on largely different DK. In detail, the H-bonding atoms play a relevant role reasonably since they encode for the presence of electrophilic groups. As seen above, aromatic rings also have a significant positive role. Notably, the presence of positively charged groups plays a marked negative role for all considered biotransformations probably because polar and ionized molecules are generally poor substrates for all metabolic reactions.

Clearly, these predictive models could be further enhanced by including stereo-electronic descriptors able to encode for the intrinsic reactivity of each atom. Nevertheless, our results emphasize the possibility of the DK to be extensively applied to the prediction of metabolic fate of a given molecule by finely recognizing the potentially reactive atoms. Further studies could also assess if DK can be similarly applied to predict the general organic reactions a given compound can undergo.

### Webserver

**Fig. 6:**
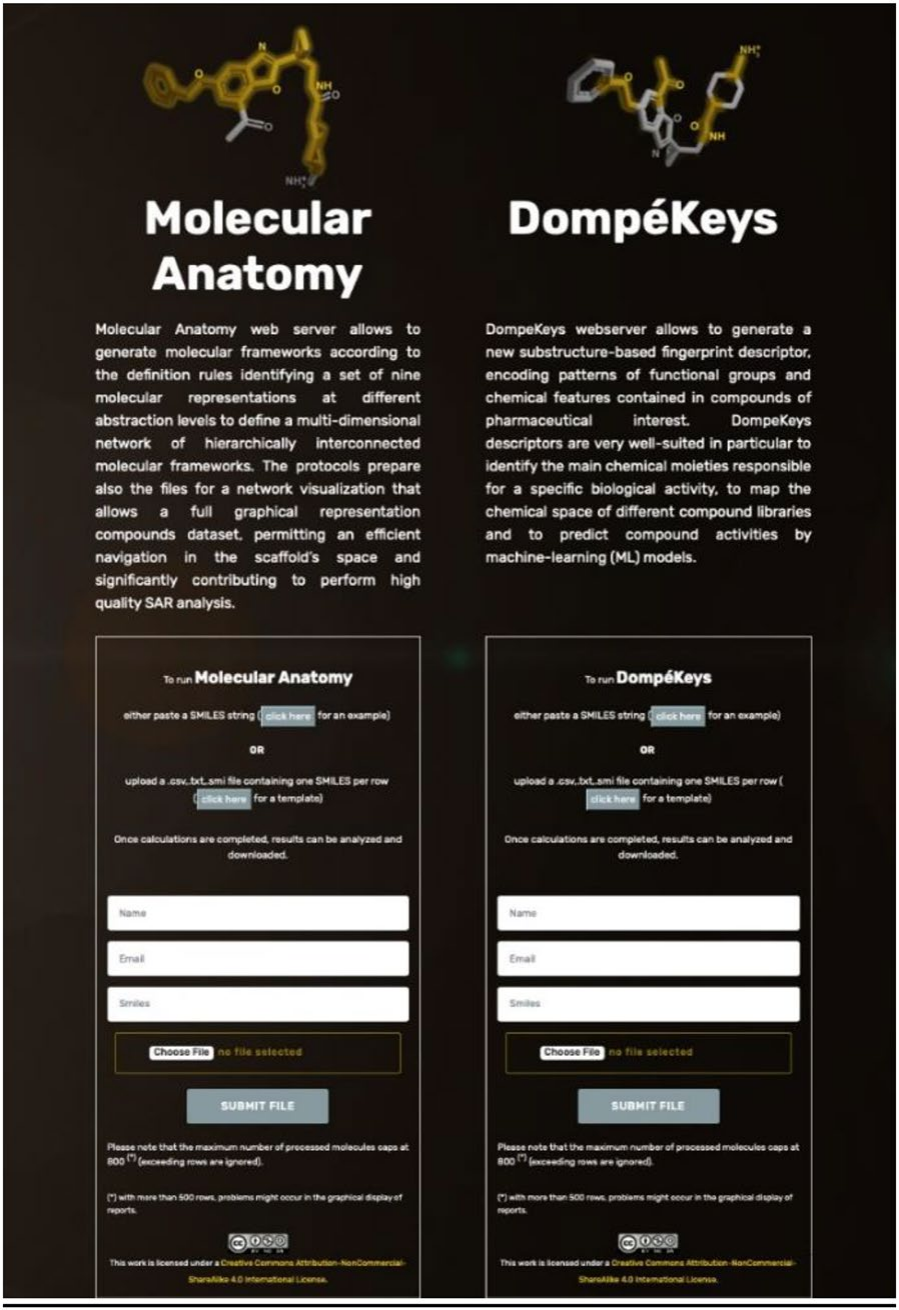
Snapshot of the webserver with the interface for DK generation

The protocol to generate the DK is publicly available within the webservice https://dompekeys.exscalate.eu (Fig. 6). The user either uploads a file containing one or more compounds, encoded as canonical SMILES, or inputs a SMILES string to generate an output table containing, for each compound (one compound for each row), the count of all the identified DK, each one reported as a separate column. Fragments corresponding to the DK present in each compound are highlighted in the molecule representation for visual analysis. The output table can be downloaded as .csv file and can be subsequently used in combination with the Molecular Anatomy approach, for the efficient analysis of compound datasets as well as for ML applications.

## Conclusions

In this work we report the DK, a substructure-based descriptor that accurately describes the key characteristics of compounds belonging to different chemical classes including, but not limited to, compounds of pharmacological interest, natural products and food components. The DK are an integral and essential part of EXSCALATE, Dompé’s end-to-end drug discovery platform. The DK are based on a comprehensive and curated list of functional groups, built using the robust SMARTS language, and organized in different levels of complexity to precisely represent molecular structures. In fact, the DK provide a very fine-grained molecular topology: for each group of interest DK also describe its chemical environment, such as the presence of aromatic or aliphatic substituents, allowing for the formulation of very precise queries. Consequently, they are very well suited to compare and assess the diversity of compound libraries, to efficiently perform substructure/similarity searches, virtual screening campaigns, and chemical space mapping. For instance, in the search of HDAC inhibitors, as illustrated in case study 1, the DK increased the hit rate of the virtual screening campaign by prioritizing compounds bearing the chemical moieties responsible for a specific biological effect, namely metal binding.

One key advantage of DK, besides their broad applicability, is that they can be rapidly precomputed and used to index large databases of compounds, whereas fingerprint-based indexing will result in redundant computations and storage space issues, and will return results that often have few or no substructures in common. By its very nature, the DK are also easily interpretable, a significant advantage in rational drug design efforts.

Lastly, DK showed adequate performance in machine learning models, predicting compounds’ chemical class and activity, in several cases outperforming other state-of-the-art descriptors as well as in predicting the occurrence of crucial metabolic reactions, namely the conjugation with glucuronic acid, the sulfate anion and glutathione, and mutagenicity. As detailed in case study 2, DK outperformed the KH descriptors in the most challenging predictions, thus proving to be well suited in recognizing the potentially reactive atoms and estimating the metabolic fate of a compound or its possible toxicity.

As a part of this study, we made freely available the full list of DK (1064 SMARTS, annotated with hierarchical levels, see Additional file 5), a Knime protocol (see Materials and Methods and Additional file 4) to generate DK, as well as a webservice at https://dompekeys.exscalate.eu, fully integrated with Dompé’s Molecular Anatomy approach for the generation and analysis of hierarchically interconnected molecular scaffolds and frameworks. With the DK approach, we go one step further by enabling clustering of molecules at different levels of chemical representation, exploiting both the scaffold-based representation encoded by Molecular Anatomy and substructure-based queries encoded by DK. Taken together, these resources enable retrieval of all most relevant information in compound libraries analysis, from both the scaffold-based representation and the functional groups identification. This provides a very thorough and integrated approach that will significantly enhance the speed and quality of the drug discovery process.

## Materials and methods

### Dataset definition

Structurally diverse compound libraries of pharmaceutical interest were used as dataset for Chemical Space Comparison Analysis. The whole dataset (Additional file 1: Table S1) collects: (i) “drugs”, including the set of safe in man drugs, commercialized or under active development in clinical phases; (ii) “peptides”, comprising di-, tri-, tetra- and pentapeptides generated by means of the VEGA suite of programs [25]; (iii) “food” and (iv) “natural” products extracted from COCONUT database [26]; (v) commercially available compounds retrieved from various sources such as ZINC [27] and eMolecules.

Diverse subsets, corresponding to the 10% of the initial datasets, were used for both commercial compounds and peptides libraries, to balance their size respect to the other dataset. In particular the subsets were generated maximizing their physico-chemical diversity by the application of the fingerprint-based Maximum Dissimilarity method, to maintain the same physico-chemical profile of the initial dataset.

Duplicates among the libraries were removed identifying overlap subsets including compounds belonging to more than a library, useful to highlight the regions of intersection in the analysis of the chemical space.

### Machine learning algorithms

Concerning the chemical space analysis, to demonstrate the capability of the different descriptor spaces to discriminate chemical classes, a multi-class classification model has been trained on the annotated library. The compounds libraries were used to train the model to discriminate a given compound’s chemical class, i.e.: peptides, natural products, food products, drugs and commercial compounds. Tree-based gradient boosting models have been trained with Knime native gradient boosting learner using default settings (i.e. number of trees = 100, learning rate = 0.1, tree depth = 20).

Regarding biological activity modelling, inhibitory data has been retrieved from ChEMBL for a set of 46 targets relevant for liability profiling during in-vitro drug discovery campaigns (Supplementary Information, Additional file 3). These targets account for a total of 7 liability types, such as: cardiotoxicity, central nervous system toxicity, gastrointestinal toxicity, endocrine disruption, pulmonary toxicity, renal toxicity and immune system toxicity.

Experimental inhibitory data has been collected from ChEMBL by UniProt identifiers. Only activity values of “IC_50_”, “EC_50_”, “K_i_ or “K_d_” measured on “human” sources have been retained. Inhibitory values were normalized to the negative log unit molar concentration and binned into two class classification problem using the cutoff of 6.5 log units (which corresponds to 300 nM). A data record above and below this cutoff has been labeled as “active” and “inactive”, respectively. This cutoff has been suggested in order to avoid class imbalanced problems and bias towards the active class [28]. Compound’s canonical SMILES notation has been used to compute molecular fingerprints.

The same above-described settings of the gradient boosting algorithm were used. Models have been validated by internal and external validation. For the former, a 70% stratified sampling has been used for train and test set definition. For the latter, fivefold cross validation (iterated 5 times) has been used. Performance comparison has been carried out by means of standard binary classification metrics, including balanced accuracy (BA), sensitivity (SE), specificity (SP), Matthews’s correlation coefficient (MCC).

The metabolism studies were based on the same datasets of first-generation metabolic reactions already utilized to develop the MetaClass tool [24]. The Kier-Hall E-states are computed by the VEGA suite of programs [25] accordingly to [29]. The models were generated by Random Forest algorithm by using Weka and applying the default settings since they provided the best performances in the previous study [23].

### Employed descriptors

DK have been benchmarked against the following fingerprint-based descriptors: (i) extended connectivity (EC) and (ii) feature invariants (FC) circular fingerprints; (iii) RDKit fingerprints (binary); (vi) MACCS keys; (v) 2D physicochemical descriptors (abbreviated as Physchem, also computed with RDKit); (vi) PubChem 881 bit structural keys. All fingerprints have been computed with a size of 1024 bits and radius of 6 (where applicable).

The public Molecular ACCess System (MACCS) structural keys [30], consisting of a dictionary of 166 pre-defined structural fragments, represent a classical descriptor in cheminformatics and were originally designed for substructure search.

The Extended-connectivity fingerprints (ECFPs) belong to the class of topological fingerprints and were specifically developed for structure–activity modeling [6]. This descriptor encodes the presence of specific circular substructures around each atom in a given molecule up to a certain bond radius. ECFPs are categorized by this parameter, in fact the maximum diameter is appended at the end of the name: ECFP4 indicates that the maximum diameter is set to 4, whereas ECFP6 denotes diameter 6. Besides the maximum diameter, the other two key parameters are the fingerprint length and identifier counts. Usually, the length of the bit string representation is kept to 1024, though a larger length reduces the possibility of bit collision. The identifier counts define if each atom identifier in an input molecule is stored only once or multiple times in case a specific substructural feature is present multiple times.

The RDKit topological fingerprints are a binary-based further implementation of the Daylight-like fingerprints in which the atom types are set based on the atomic number and aromaticity (RDKit: Cheminformatics and Machine Learning Software. http://www.rdkit.org). The PubChem 881 structural key is a 881-bit-long fingerprint implemented in PubChem for similarity search and neighboring (ftp://ftp.ncbi.nlm.nih.gov/pubchem/specifications/pubchem_fingerprints.pdf).

### Validation protocol

A Pipeline Pilot protocol was implemented to validate the ability of DK to correctly map structural moieties and pharmacophoric features. In particular two steps of validation were applied, the first one to verify that the DKs corresponding to structural moieties (level 0 and 1) were able to retrieve themselves and that no duplicates are found within each level. The validation process was thus iterated on the DKs of level 0 and 1, by converting them in whole molecules and performing, in parallel, substructure searches of both the single corresponding DK and of the entire list of DKs. This procedure allows to visually analyze the molecular structure corresponding to a given DK and to verify the correctness of each SMARTS string. Moreover, it enables to analyze molecules mapping more than one DK, excluding overlaps and demonstrating the complementarity between structural information encoded by DKs belonging to different levels. The second step of validation involved DK from level 3 and 2. All possible permutations (filling the molecule free valences with H, methyl and aromatic ring) for each generic SMARTS, conversion into molecules, and substructure search (using as query both the generic SMARTS of level 3 and the more specific SMARTS of level 2) were accomplished by using a custom pilot script.

### Web interface implementation

The web interface was implemented using LAMP (Linux Apache MariaDB PHP), an open-source web development platform enabling optimal performances in displaying and handling the user’s input and output data. The DK are calculated on the fly through an underlying, completely automated Pipeline Pilot workflow.

### Knime implementation

A protocol was implemented in Knime [31] to carry out DK calculation for an input file of compounds in SMILES format. As an example, the ChEMBL dataset employed for biological activity modelling was used as input. The protocol performs DK calculation and count on the basis of a curated list of 77 representative SMARTS, selected among the most relevant chemical classes and covering all the hierarchical levels.

### Supplementary Information

**Additional file 1: Table S1.** Compound libraries used in Chemical Space Comparison Analysis. **Table S2. **Descriptors performance in chemical space classification. **Table S3. **Descriptors performance in biological activity modelling. **Table S4. **MCC performances as a function of the DK descriptor subsets. **Table S5.** Activity thresholds for 26,092 commercial compounds tested at 10 μM concentration against HDAC7, classified on the basis of enzyme activity percent of inhibition. **Figure S1**. Schematization of the results of decision tree based on the descriptors characterized by (A) a broader definition of functional groups (e.g. amine derivatives) by DK level 3, and (B) a more precise mapping of a given functional group (e.g. a tertiary amine with aliphatic substituents) by DK level 2. **Figure S2.** Frequency distribution of DK on ChEMBL datasets.

**Additional file 2: **Validation protocol and example file to run the protocol.

**Additional file 3: **Data set employed for biological activity modelling.

**Additional file 4: **Knime protocol for DK calculation.

**Additional file 5: **The full list of DK.

**Additional file 6: **List of DK and their frequency among hERG inhibitors.

## Data Availability

The relevant data supporting the conclusions of this work are available in this published article and its Additional files.
