# Supplementary material for: “DompeKeys”: a set of novel substructure-based descriptors for efficient chemical space mapping, development and structural interpretation of machine learning models, and indexing of large databases
Source: J Cheminform. 2024 Feb 23;16:21. doi: 10.1186/s13321-024-00813-4 (PMC10893756; doi:10.1186/s13321-024-00813-4)
Supplement: Supplementary file 1 — Additional file 1: Table S1. Compound libraries used in Chemical Space Comparison Analysis. Table S2. Descriptors performance in chemical space classification. Table S3. Descriptors performance in biological activity modelling. Table S4. MCC performances as a function of the DK descriptor subsets. Table S5. Activity thresholds for 26,092 commercial compounds tested at 10 μM concentration against HDAC7, classified on the basis of enzyme activity percent of inhibition. Figure S1. Schematization of the results of decision tree based on the descriptors characterized by (A) a broader definition of functional groups (e.g. amine derivatives) by DK level 3, and (B) a more precise mapping of a given functional group (e.g. a tertiary amine with aliphatic substituents) by DK level 2. Figure S2. Frequency distribution of DK on ChEMBL datasets. [file 13321_2024_813_MOESM1_ESM.docx]

**Additional file**

**“DompeKeys”: a set of novel substructure-based descriptors for efficient chemical space mapping, development and structural interpretation of machine learning models, and indexing of large databases.**

Candida Manelfi^1^*, Valerio Tazzari^1^*, Filippo Lunghini^1^, Carmen Cerchia^2^, Anna Fava^1^, Alessandro Pedretti^3^, Pieter F.W. Stouten^1,4^, Giulio Vistoli^3^ and Andrea Rosario Beccari^1^**

*first author

**Correspondence: andrea.beccari@dompe.com

^1^ EXSCALATE, Dompé Farmaceutici SpA, Via Tommaso de Amicis 95, Napoli 80123, Italy.

^2^ Department of Pharmacy, University of Naples “Federico II”, Via D. Montesano 49, 80131 Napoli, Italy.

^3^ Dipartimento di Scienze Farmaceutiche, Università degli Studi di Milano, Via Mangiagalli, 25, I-20133 Milano, Italy.

^4^ Stouten Pharma Consultancy BV, Kempenarestraat 47, 2860 Sint-Katelijne-Waver, Belgium.

**Table S1.** Compound libraries used in Chemical Space Comparison Analysis. The following properties are used to characterize the molecules: Molecular weight (MW); the octanol/water partition coefficient (Log P) calculated using the atom-based method published by Ghose and Crippen; Polar Surface Area (PSA) calculated using a 2D approximation; number of rotational groups (N rotors).

| Library | n. molecules | MW (g/mol) | Log P | PSA (Å^2^) | N rotors |
| --- | --- | --- | --- | --- | --- |
| Commercial Compounds | 4,964,529 | 355.13 ± 49.78 | 2.72 ± 1.64 | 79.06 ± 27.48 | 5.05 ± 2.06 |
| Natural compounds (NC) | 263,529 | 435.33 ±186.15 | 3.10 ± 2.89 | 101.77 ± 76.82 | 6.35 ± 6.1 |
| Drugs (DRG) | 8,721 | 414.84 ± 183.4 | 2.55 ± 2.94 | 101.81 ± 76.04 | 6.87 ± 5.66 |
| Foods (FOOD) | 65,461 | 722.39 ± 262.73 | 14.62 ± 8.59 | 93.31 ± 63.66 | 37.91 ± 20.45 |
| Dipeptides (2PEP) | 400 | 296.84 ± 42.6 | -1.50 ± 1.33 | 142.46 ± 27.18 | 7.60 ±1.77 |
| Tripeptides (3PEP) | 8,000 | 415.72 ± 52.10 | -1.87 ± 1.64 | 192.14 ± 33.25 | 11.40 ± 2.16 |
| Tetrapeptides (4PEP) | 160,000 | 534.61 ± 60.16 | -2.25 ± 1.92 | 241.82 ± 38.39 | 15.2 ± 2.51 |
| Pentapeptides (5PEP) | 3,200,000 | 653.49 ± 67.26 | -2.64 ± 2.18 | 291.50 ± 42.92 | 19 ± 2.79 |

**Table S2.** Descriptors performance in chemical space classification.

| **Descriptor** | **Library** | **Balanced Accuracy** | **Recall** | **Precision** | **Sensitivity** | **Specificity** | **F-measure** |
| --- | --- | --- | --- | --- | --- | --- | --- |
| **DOMPE KEYS** | **Commercial Compounds** | **0.86** | **0.74** | **0.94** | **0.74** | **0.97** | **0.83** |
|  | **Drugs** | **0.83** | **0.75** | **0.01** | **0.75** | **0.92** | **0.03** |
|  | **Food Products** | **0.90** | **0.83** | **0.76** | **0.83** | **0.98** | **0.79** |
|  | **Natural Products** | **0.87** | **0.79** | **0.82** | **0.79** | **0.94** | **0.81** |
|  | **Peptides** | **1.00** | **1.00** | **1.00** | **1.00** | **1.00** | **1.00** |
| **EC** | **Commercial Compounds** | **0.83** | **0.71** | **0.89** | **0.71** | **0.95** | **0.79** |
|  | **Drugs** | **0.74** | **0.58** | **0.01** | **0.58** | **0.91** | **0.02** |
|  | **Food Products** | **0.89** | **0.78** | **0.82** | **0.78** | **0.99** | **0.80** |
|  | **Natural Products** | **0.84** | **0.74** | **0.79** | **0.74** | **0.94** | **0.77** |
|  | **Peptides** | **1.00** | **1.00** | **1.00** | **1.00** | **1.00** | **1.00** |
| **FC** | **Commercial Compounds** | **0.84** | **0.73** | **0.89** | **0.73** | **0.94** | **0.80** |
|  | **Drugs** | **0.76** | **0.61** | **0.01** | **0.61** | **0.91** | **0.02** |
|  | **Food Products** | **0.89** | **0.80** | **0.80** | **0.80** | **0.99** | **0.80** |
|  | **Natural Products** | **0.84** | **0.73** | **0.80** | **0.73** | **0.94** | **0.76** |
|  | **Peptides** | **1.00** | **1.00** | **1.00** | **1.00** | **1.00** | **1.00** |
| **MACCS** | **Commercial Compounds** | **0.86** | **0.76** | **0.93** | **0.76** | **0.97** | **0.83** |
|  | **Drugs** | **0.81** | **0.71** | **0.01** | **0.71** | **0.92** | **0.03** |
|  | **Food Products** | **0.90** | **0.83** | **0.75** | **0.83** | **0.98** | **0.79** |
|  | **Natural Products** | **0.86** | **0.78** | **0.84** | **0.78** | **0.95** | **0.81** |
|  | **Peptides** | **1.00** | **1.00** | **1.00** | **1.00** | **1.00** | **1.00** |
| **RDKIT** | **Commercial Compounds** | **0.88** | **0.80** | **0.91** | **0.80** | **0.95** | **0.85** |
|  | **Drugs** | **0.76** | **0.60** | **0.01** | **0.60** | **0.92** | **0.03** |
|  | **Food Products** | **0.91** | **0.83** | **0.72** | **0.83** | **0.98** | **0.77** |
|  | **Natural Products** | **0.84** | **0.72** | **0.86** | **0.72** | **0.96** | **0.78** |
|  | **Peptides** | **1.00** | **1.00** | **1.00** | **1.00** | **1.00** | **1.00** |
| **PubChem** | **Commercial Compounds** | **0.87** | **0.76** | **0.94** | **0.76** | **0.97** | **0.84** |
|  | **Drugs** | **0.83** | **0.75** | **0.02** | **0.75** | **0.92** | **0.03** |
|  | **Food Products** | **0.91** | **0.83** | **0.75** | **0.83** | **0.98** | **0.79** |
|  | **Natural Products** | **0.87** | **0.79** | **0.84** | **0.79** | **0.95** | **0.82** |
|  | **Peptides** | **1.00** | **1.00** | **1.00** | **1.00** | **1.00** | **1.00** |

**Table S3.** Descriptors performance in biological activity modelling**.**

| **target** | **DK** | | | | **EC** | | | | **FC** | | | | **MACCS** | | | | | **RDKIT** | | | | | **PUBCHEM** | | | | |
| --- | --- | --- | --- | --- | --- | --- | --- | --- | --- | --- | --- | --- | --- | --- | --- | --- | --- | --- | --- | --- | --- | --- | --- | --- | --- | --- | --- |
|  | **BA** | **SE** | **SP** | **MCC** | **BA*** | **SE*** | **SP*** | **MCC*** | **BA*** | **SE** | **SP*** | **MCC*** | **BA** | **SE*** | **SP*** | **MCC*** | **BA*** | | **SE*** | **SP*** | **MCC*** | **BA*** | | **SE** | **SP*** | **MCC*** |  |
| ACHE | 0.83 | 0.86 | 0.8 | 0.67 | 0.8 | 0.8 | 0.8 | 0.6 | 0.78 | 0.83 | 0.74 | 0.56 | 0.82 | 0.83 | 0.81 | 0.64 | 0.8 | | 0.8 | 0.79 | 0.59 | 0.82 | | 0.84 | 0.81 | 0.65 |  |
| ADORA1 | 0.8 | 0.83 | 0.77 | 0.59 | 0.79 | 0.82 | 0.75 | 0.58 | 0.79 | 0.81 | 0.76 | 0.57 | 0.73 | 0.77 | 0.7 | 0.47 | 0.74 | | 0.78 | 0.71 | 0.49 | 0.74 | | 0.77 | 0.71 | 0.48 |  |
| ADORA2A | 0.77 | 0.88 | 0.67 | 0.57 | 0.74 | 0.85 | 0.62 | 0.49 | 0.78 | 0.88 | 0.67 | 0.57 | 0.74 | 0.82 | 0.66 | 0.49 | 0.76 | | 0.86 | 0.66 | 0.54 | 0.76 | | 0.85 | 0.66 | 0.52 |  |
| ADRA1A | 0.64 | 0.91 | 0.37 | 0.31 | 0.69 | 0.91 | 0.46 | 0.4 | 0.6 | 0.89 | 0.32 | 0.23 | 0.7 | 0.88 | 0.51 | 0.39 | 0.71 | | 0.9 | 0.51 | 0.42 | 0.71 | | 0.89 | 0.52 | 0.42 |  |
| ADRA2A | 0.73 | 0.81 | 0.66 | 0.48 | 0.79 | 0.79 | 0.79 | 0.58 | 0.73 | 0.76 | 0.7 | 0.46 | 0.68 | 0.68 | 0.68 | 0.36 | 0.79 | | 0.81 | 0.77 | 0.57 | 0.79 | | 0.83 | 0.75 | 0.58 |  |
| ADRA2B | 0.74 | 0.67 | 0.82 | 0.49 | 0.66 | 0.62 | 0.69 | 0.3 | 0.58 | 0.52 | 0.64 | 0.16 | 0.71 | 0.67 | 0.74 | 0.4 | 0.82 | | 0.76 | 0.87 | 0.63 | 0.75 | | 0.7 | 0.79 | 0.48 |  |
| ADRB1 | 0.71 | 0.82 | 0.61 | 0.43 | 0.7 | 0.79 | 0.61 | 0.39 | 0.73 | 0.81 | 0.66 | 0.47 | 0.69 | 0.79 | 0.59 | 0.38 | 0.7 | | 0.8 | 0.61 | 0.41 | 0.71 | | 0.82 | 0.61 | 0.43 |  |
| ADRB2 | 0.75 | 0.88 | 0.62 | 0.5 | 0.82 | 0.94 | 0.69 | 0.66 | 0.8 | 0.92 | 0.67 | 0.61 | 0.8 | 0.93 | 0.67 | 0.62 | 0.85 | | 0.92 | 0.77 | 0.68 | 0.81 | | 0.92 | 0.71 | 0.63 |  |
| AR | 0.75 | 0.89 | 0.61 | 0.53 | 0.7 | 0.83 | 0.56 | 0.41 | 0.7 | 0.86 | 0.53 | 0.43 | 0.74 | 0.84 | 0.65 | 0.5 | 0.67 | | 0.79 | 0.54 | 0.35 | 0.75 | | 0.81 | 0.69 | 0.51 |  |
| AVPR1A | 0.72 | 0.84 | 0.6 | 0.45 | 0.74 | 0.86 | 0.62 | 0.49 | 0.72 | 0.82 | 0.62 | 0.44 | 0.66 | 0.79 | 0.53 | 0.33 | 0.75 | | 0.83 | 0.66 | 0.49 | 0.72 | | 0.84 | 0.61 | 0.45 |  |
| CCKAR | 0.86 | 0.84 | 0.88 | 0.72 | 0.86 | 0.82 | 0.9 | 0.72 | 0.87 | 0.89 | 0.85 | 0.74 | 0.9 | 0.89 | 0.9 | 0.79 | 0.85 | | 0.84 | 0.85 | 0.7 | 0.8 | | 0.8 | 0.8 | 0.59 |  |
| CHRM1 | 0.78 | 0.82 | 0.73 | 0.56 | 0.67 | 0.73 | 0.61 | 0.34 | 0.69 | 0.79 | 0.59 | 0.4 | 0.71 | 0.79 | 0.63 | 0.43 | 0.71 | | 0.79 | 0.63 | 0.43 | 0.74 | | 0.78 | 0.7 | 0.48 |  |
| CHRM3 | 0.84 | 0.85 | 0.83 | 0.65 | 0.77 | 0.78 | 0.75 | 0.51 | 0.82 | 0.81 | 0.83 | 0.6 | 0.74 | 0.81 | 0.68 | 0.47 | 0.8 | | 0.83 | 0.77 | 0.59 | 0.79 | | 0.87 | 0.72 | 0.58 |  |
| CNR1 | 0.73 | 0.83 | 0.63 | 0.47 | 0.74 | 0.82 | 0.66 | 0.49 | 0.73 | 0.83 | 0.63 | 0.48 | 0.72 | 0.82 | 0.63 | 0.46 | 0.74 | | 0.81 | 0.67 | 0.49 | 0.76 | | 0.84 | 0.68 | 0.53 |  |
| CNR2 | 0.78 | 0.86 | 0.7 | 0.57 | 0.78 | 0.91 | 0.65 | 0.58 | 0.79 | 0.92 | 0.66 | 0.6 | 0.75 | 0.81 | 0.69 | 0.5 | 0.73 | | 0.83 | 0.63 | 0.47 | 0.76 | | 0.87 | 0.65 | 0.53 |  |
| CYP19A1 | 0.72 | 0.73 | 0.72 | 0.45 | 0.68 | 0.73 | 0.63 | 0.36 | 0.69 | 0.69 | 0.68 | 0.37 | 0.73 | 0.76 | 0.7 | 0.47 | 0.72 | | 0.75 | 0.7 | 0.45 | 0.76 | | 0.8 | 0.72 | 0.53 |  |
| DRD1 | 0.83 | 0.84 | 0.82 | 0.66 | 0.82 | 0.84 | 0.8 | 0.64 | 0.85 | 0.83 | 0.88 | 0.7 | 0.78 | 0.84 | 0.73 | 0.57 | 0.82 | | 0.89 | 0.76 | 0.65 | 0.76 | | 0.78 | 0.73 | 0.51 |  |
| DRD2 | 0.75 | 0.86 | 0.64 | 0.51 | 0.81 | 0.89 | 0.73 | 0.62 | 0.79 | 0.86 | 0.72 | 0.58 | 0.73 | 0.82 | 0.63 | 0.46 | 0.74 | | 0.84 | 0.65 | 0.5 | 0.76 | | 0.84 | 0.67 | 0.52 |  |
| EDNRA | 0.73 | 0.87 | 0.58 | 0.47 | 0.63 | 0.68 | 0.58 | 0.24 | 0.6 | 0.77 | 0.42 | 0.19 | 0.72 | 0.77 | 0.67 | 0.41 | 0.72 | | 0.77 | 0.67 | 0.41 | 0.73 | | 0.84 | 0.63 | 0.46 |  |
| ESR1 | 0.76 | 0.84 | 0.69 | 0.53 | 0.74 | 0.81 | 0.67 | 0.48 | 0.78 | 0.86 | 0.7 | 0.56 | 0.74 | 0.8 | 0.69 | 0.49 | 0.77 | | 0.84 | 0.7 | 0.55 | 0.8 | | 0.85 | 0.76 | 0.6 |  |
| HRH1 | 0.73 | 0.84 | 0.62 | 0.47 | 0.7 | 0.81 | 0.59 | 0.4 | 0.7 | 0.82 | 0.59 | 0.42 | 0.72 | 0.81 | 0.62 | 0.44 | 0.68 | | 0.82 | 0.53 | 0.37 | 0.74 | | 0.85 | 0.64 | 0.5 |  |
| HRH2 | 0.74 | 0.73 | 0.75 | 0.45 | 0.75 | 0.8 | 0.69 | 0.45 | 0.72 | 0.67 | 0.78 | 0.42 | 0.74 | 0.73 | 0.75 | 0.45 | 0.68 | | 0.53 | 0.83 | 0.37 | 0.73 | | 0.63 | 0.82 | 0.45 |  |
| HTR1A | 0.74 | 0.89 | 0.6 | 0.49 | 0.72 | 0.9 | 0.53 | 0.46 | 0.75 | 0.88 | 0.63 | 0.5 | 0.73 | 0.87 | 0.6 | 0.47 | 0.71 | | 0.87 | 0.56 | 0.43 | 0.71 | | 0.9 | 0.53 | 0.45 |  |
| HTR1B | 0.76 | 0.91 | 0.61 | 0.54 | 0.68 | 0.88 | 0.48 | 0.37 | 0.75 | 0.94 | 0.57 | 0.55 | 0.76 | 0.91 | 0.61 | 0.54 | 0.72 | | 0.93 | 0.52 | 0.49 | 0.76 | | 0.89 | 0.63 | 0.52 |  |
| HTR2A | 0.75 | 0.87 | 0.63 | 0.51 | 0.7 | 0.87 | 0.53 | 0.42 | 0.74 | 0.86 | 0.62 | 0.49 | 0.69 | 0.79 | 0.59 | 0.38 | 0.71 | | 0.82 | 0.59 | 0.42 | 0.74 | | 0.86 | 0.63 | 0.49 |  |
| HTR2B | 0.66 | 0.75 | 0.58 | 0.33 | 0.66 | 0.8 | 0.52 | 0.34 | 0.68 | 0.78 | 0.58 | 0.37 | 0.63 | 0.69 | 0.57 | 0.26 | 0.65 | | 0.75 | 0.56 | 0.31 | 0.65 | | 0.71 | 0.6 | 0.31 |  |
| HTR3A | 0.8 | 0.92 | 0.68 | 0.62 | 0.71 | 0.84 | 0.58 | 0.42 | 0.7 | 0.85 | 0.55 | 0.41 | 0.76 | 0.9 | 0.63 | 0.55 | 0.71 | | 0.85 | 0.58 | 0.43 | 0.76 | | 0.86 | 0.67 | 0.53 |  |
| KCNA5 | 0.65 | 0.67 | 0.63 | 0.3 | 0.66 | 0.63 | 0.7 | 0.33 | 0.5 | 0.33 | 0.67 | 0 | 0.59 | 0.63 | 0.56 | 0.18 | 0.55 | | 0.54 | 0.56 | 0.1 | 0.59 | | 0.55 | 0.64 | 0.19 |  |
| KCNH2 | 0.77 | 0.57 | 0.97 | 0.6 | 0.7 | 0.5 | 0.9 | 0.39 | 0.74 | 0.58 | 0.9 | 0.45 | 0.71 | 0.52 | 0.91 | 0.42 | 0.75 | | 0.59 | 0.91 | 0.49 | 0.75 | | 0.55 | 0.95 | 0.52 |  |
| LCK | 0.77 | 0.89 | 0.65 | 0.56 | 0.69 | 0.83 | 0.56 | 0.4 | 0.74 | 0.74 | 0.74 | 0.47 | 0.77 | 0.86 | 0.67 | 0.55 | 0.77 | | 0.86 | 0.67 | 0.55 | 0.71 | | 0.79 | 0.63 | 0.41 |  |
| MAOA | 0.72 | 0.52 | 0.93 | 0.48 | 0.77 | 0.66 | 0.89 | 0.53 | 0.76 | 0.66 | 0.87 | 0.49 | 0.77 | 0.66 | 0.88 | 0.51 | 0.7 | | 0.59 | 0.81 | 0.35 | 0.77 | | 0.66 | 0.89 | 0.52 |  |
| NR3C1 | 0.72 | 0.94 | 0.51 | 0.48 | 0.63 | 0.88 | 0.38 | 0.25 | 0.67 | 0.89 | 0.44 | 0.34 | 0.7 | 0.94 | 0.46 | 0.46 | 0.7 | | 0.9 | 0.49 | 0.4 | 0.74 | | 0.91 | 0.58 | 0.48 |  |
| OPRD1 | 0.81 | 0.9 | 0.73 | 0.65 | 0.8 | 0.84 | 0.76 | 0.59 | 0.77 | 0.82 | 0.73 | 0.55 | 0.81 | 0.89 | 0.73 | 0.63 | 0.81 | | 0.86 | 0.76 | 0.62 | 0.83 | | 0.88 | 0.78 | 0.66 |  |
| OPRK1 | 0.81 | 0.83 | 0.78 | 0.61 | 0.8 | 0.87 | 0.73 | 0.61 | 0.8 | 0.88 | 0.73 | 0.62 | 0.78 | 0.83 | 0.73 | 0.55 | 0.74 | | 0.79 | 0.69 | 0.48 | 0.79 | | 0.85 | 0.72 | 0.58 |  |
| OPRM1 | 0.78 | 0.87 | 0.7 | 0.57 | 0.78 | 0.85 | 0.71 | 0.56 | 0.8 | 0.88 | 0.71 | 0.6 | 0.78 | 0.85 | 0.71 | 0.57 | 0.76 | | 0.85 | 0.68 | 0.53 | 0.78 | | 0.86 | 0.7 | 0.56 |  |
| PDE3A | 0.89 | 0.82 | 0.96 | 0.8 | 0.78 | 0.82 | 0.75 | 0.53 | 0.76 | 0.82 | 0.71 | 0.49 | 0.83 | 0.82 | 0.83 | 0.63 | 0.89 | | 0.91 | 0.88 | 0.75 | 0.8 | | 0.74 | 0.86 | 0.59 |  |
| PDE4D | 0.85 | 0.89 | 0.8 | 0.7 | 0.78 | 0.86 | 0.71 | 0.58 | 0.84 | 0.95 | 0.73 | 0.71 | 0.82 | 0.83 | 0.8 | 0.64 | 0.79 | | 0.89 | 0.69 | 0.6 | 0.82 | | 0.85 | 0.79 | 0.64 |  |
| PPARA | 0.73 | 0.68 | 0.78 | 0.46 | 0.73 | 0.67 | 0.79 | 0.45 | 0.74 | 0.7 | 0.78 | 0.48 | 0.76 | 0.75 | 0.77 | 0.51 | 0.78 | | 0.8 | 0.75 | 0.54 | 0.73 | | 0.68 | 0.78 | 0.45 |  |
| PPARD | 0.83 | 0.91 | 0.76 | 0.68 | 0.79 | 0.77 | 0.8 | 0.56 | 0.75 | 0.88 | 0.61 | 0.52 | 0.82 | 0.83 | 0.81 | 0.64 | 0.82 | | 0.88 | 0.76 | 0.65 | 0.8 | | 0.85 | 0.74 | 0.6 |  |
| PPARG | 0.81 | 0.84 | 0.78 | 0.63 | 0.77 | 0.76 | 0.77 | 0.53 | 0.73 | 0.75 | 0.71 | 0.46 | 0.75 | 0.75 | 0.76 | 0.51 | 0.78 | | 0.81 | 0.76 | 0.56 | 0.79 | | 0.8 | 0.78 | 0.58 |  |
| PTGS1 | 0.71 | 0.5 | 0.91 | 0.39 | 0.64 | 0.3 | 0.97 | 0.37 | 0.74 | 0.6 | 0.88 | 0.42 | 0.71 | 0.5 | 0.93 | 0.43 | 0.69 | | 0.5 | 0.88 | 0.34 | 0.72 | | 0.5 | 0.93 | 0.44 |  |
| PTGS2 | 0.77 | 0.74 | 0.79 | 0.53 | 0.73 | 0.73 | 0.73 | 0.45 | 0.8 | 0.76 | 0.83 | 0.59 | 0.71 | 0.68 | 0.75 | 0.42 | 0.75 | | 0.76 | 0.74 | 0.49 | 0.78 | | 0.75 | 0.82 | 0.56 |  |
| SCN5A | 0.72 | 0.55 | 0.9 | 0.42 | 0.63 | 0.36 | 0.9 | 0.27 | 0.69 | 0.45 | 0.92 | 0.37 | 0.77 | 0.64 | 0.9 | 0.49 | 0.83 | | 0.73 | 0.93 | 0.61 | 0.69 | | 0.47 | 0.9 | 0.35 |  |
| SLC6A2 | 0.76 | 0.9 | 0.62 | 0.55 | 0.71 | 0.79 | 0.64 | 0.43 | 0.76 | 0.9 | 0.62 | 0.56 | 0.76 | 0.87 | 0.64 | 0.53 | 0.76 | | 0.89 | 0.62 | 0.54 | 0.79 | | 0.87 | 0.71 | 0.58 |  |
| SLC6A3 | 0.79 | 0.8 | 0.77 | 0.57 | 0.8 | 0.82 | 0.78 | 0.6 | 0.77 | 0.81 | 0.73 | 0.54 | 0.8 | 0.8 | 0.81 | 0.61 | 0.77 | | 0.78 | 0.76 | 0.53 | 0.79 | | 0.79 | 0.78 | 0.57 |  |
| SLC6A4 | 0.8 | 0.92 | 0.68 | 0.62 | 0.8 | 0.92 | 0.67 | 0.62 | 0.81 | 0.93 | 0.69 | 0.65 | 0.75 | 0.87 | 0.63 | 0.51 | 0.77 | | 0.92 | 0.61 | 0.57 | 0.77 | | 0.89 | 0.65 | 0.56 |  |

* means that models built on DK are significantly better (p-value < 0.05 at 95%) than the compared descriptor for the given performance metric.

**Table S4.** MCC performances as a function of the DK descriptor subsets.

| **Included DK levels** | **Average MCC over ChEMBL datasets (stdev)** |
| --- | --- |
| 0, 1 | 0.48 (0.11) |
| 2 | 0.48 (0.13) |
| 3 | 0.44 (0.12) |
| 4 | 0.40 (0.13) |
| 0, 1, 2, 3, 4 | 0.54 (0.10) |

**Table S5.** Activity thresholds for 26,092 commercial compounds tested at 10 μM concentration against HDAC7, classified on the basis of enzyme activity percent of inhibition.

| **Activity class** | **N. of Compounds** | **% of Inhibition** |
| --- | --- | --- |
| Inactive | 23750 | < 19 |
| Weak | 2141 | 19–33 |
| Moderate | 144 | 33–50 |
| Strong | 37 | 50–80 |
| Very strong | 20 | > 80 |


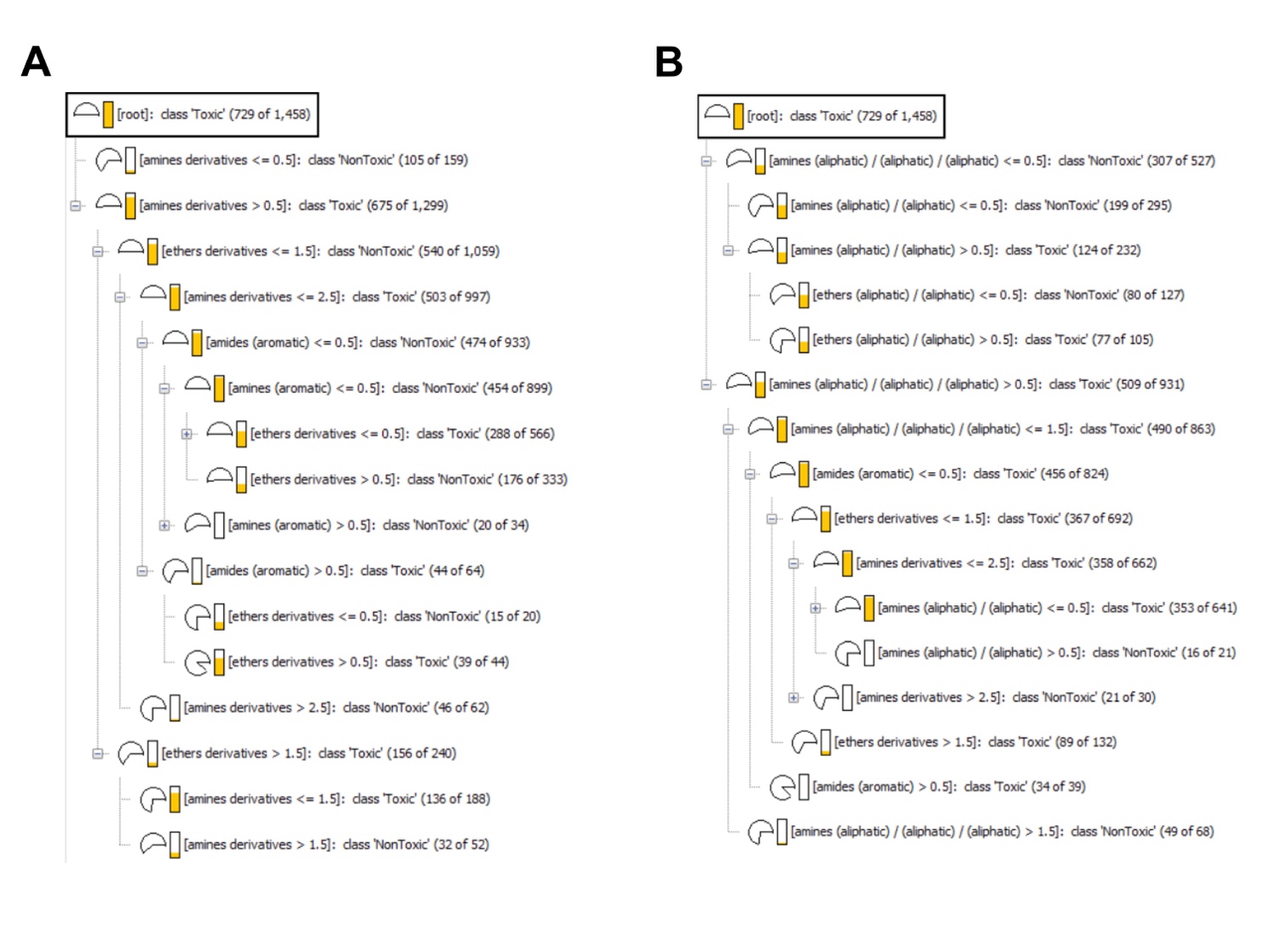


**Figure S1**. Schematization of the results of decision tree based on the descriptors characterized by (A) a broader definition of functional groups (e.g. amine derivatives) by DK level 3, and (B) a more precise mapping of a given functional group (e.g. a tertiary amine with aliphatic substituents) by DK level 2.


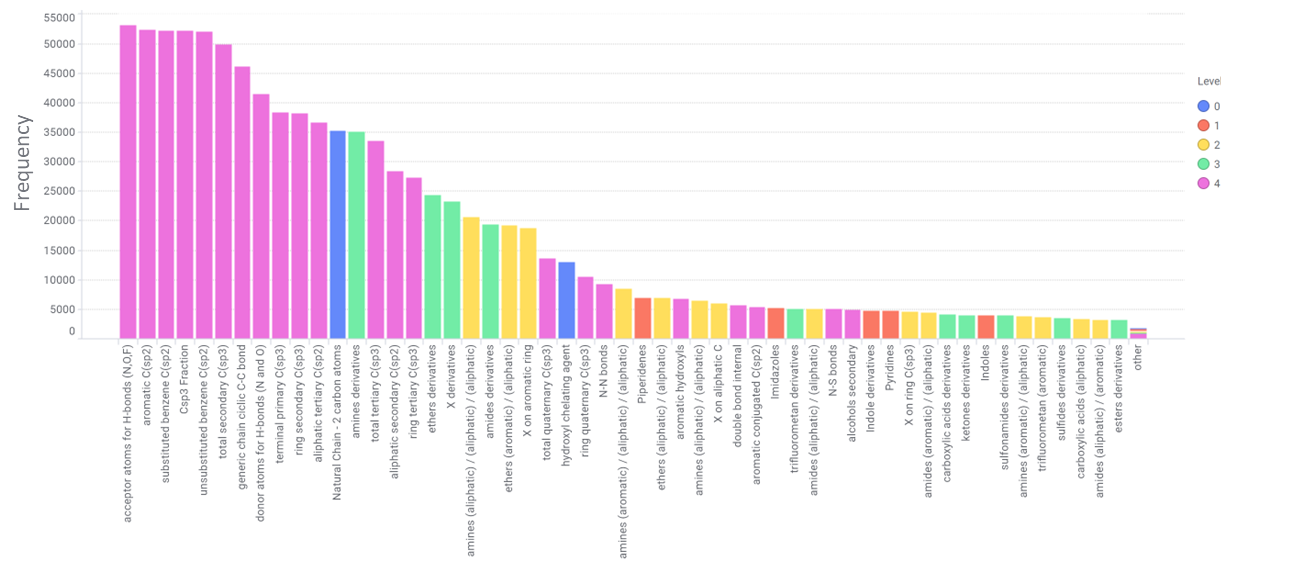


**Figure S2**. Frequency distribution of DK on ChEMBL datasets.
